# Supplementary figures and images for: Distinct patterns of connectivity with the motor cortex reflect different components of sensorimotor learning
Source: PLoS Biol. 2024 Dec 3;22(12):e3002934. doi: 10.1371/journal.pbio.3002934 (PMC11644839; doi:10.1371/journal.pbio.3002934)

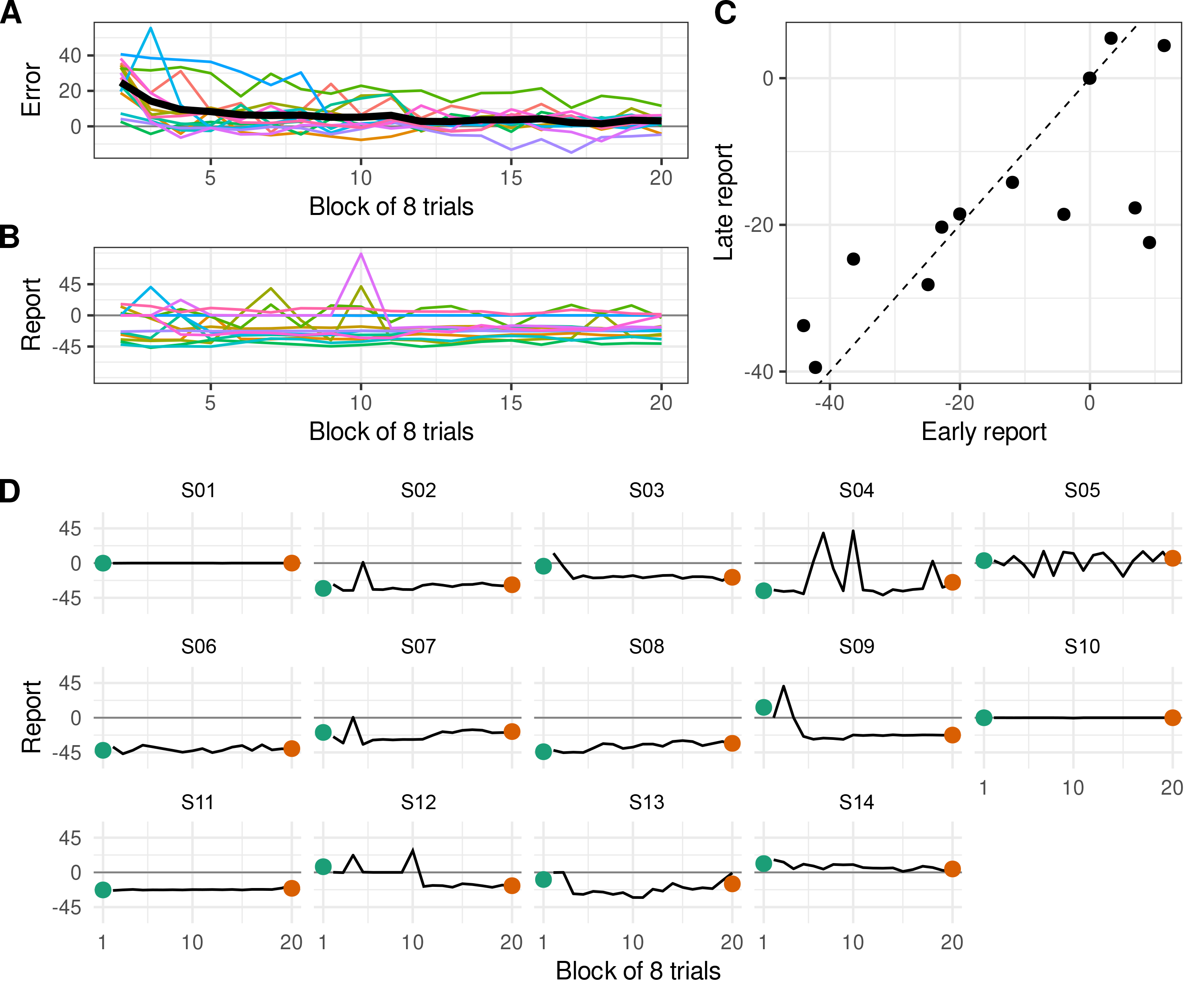

Supplement: S1 Fig — (TIF) [file pbio.3002934.s001.tif]

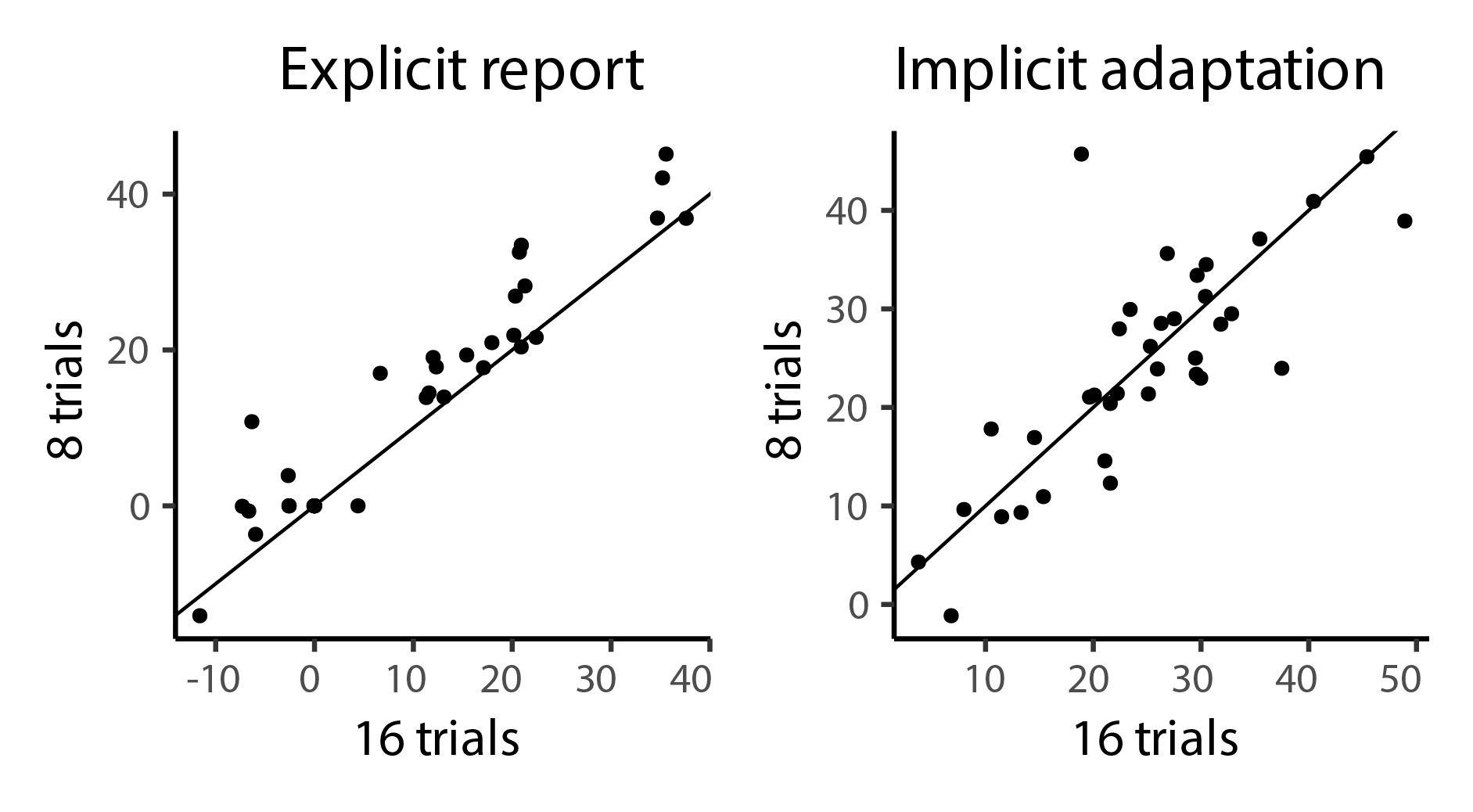

Supplement: S2 Fig — (TIF) [file pbio.3002934.s002.tif]

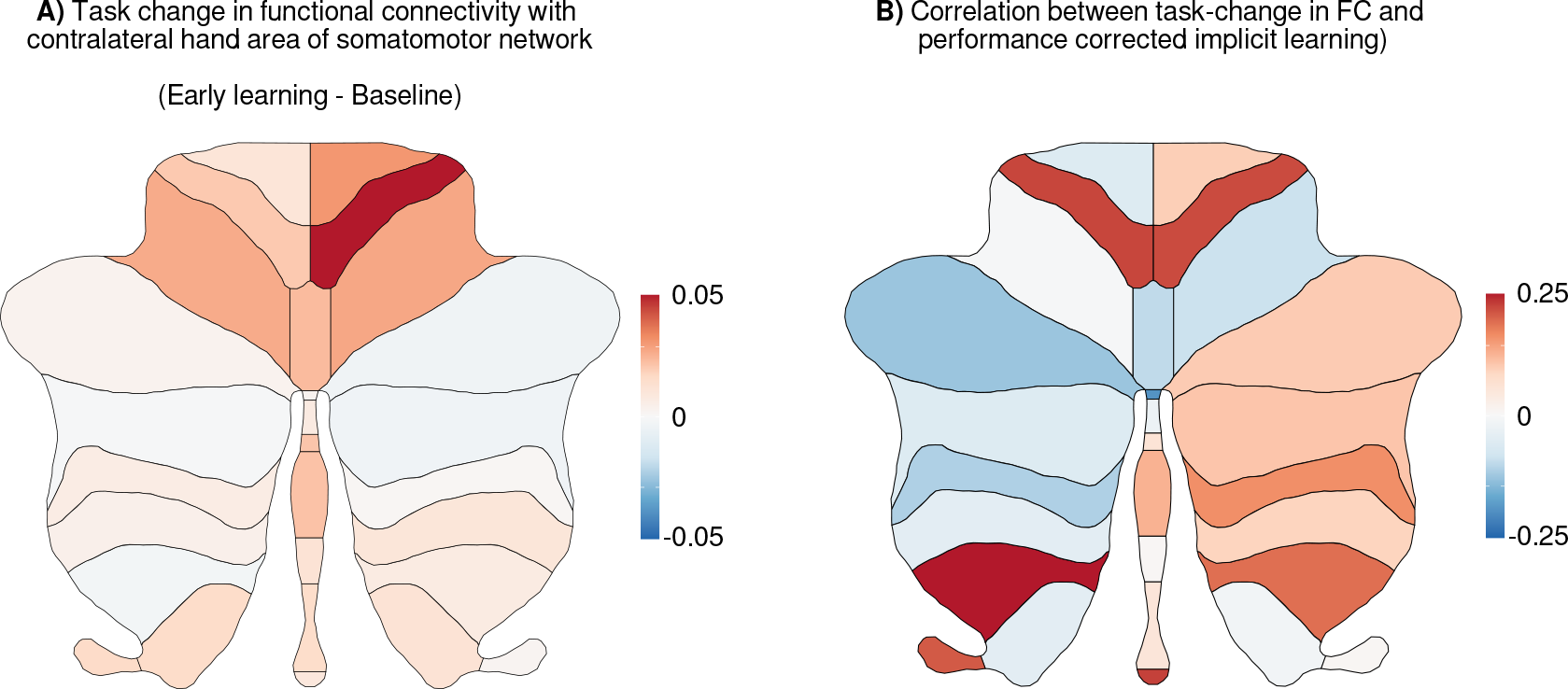

Supplement: S3 Fig — (TIF) [file pbio.3002934.s003.tif]

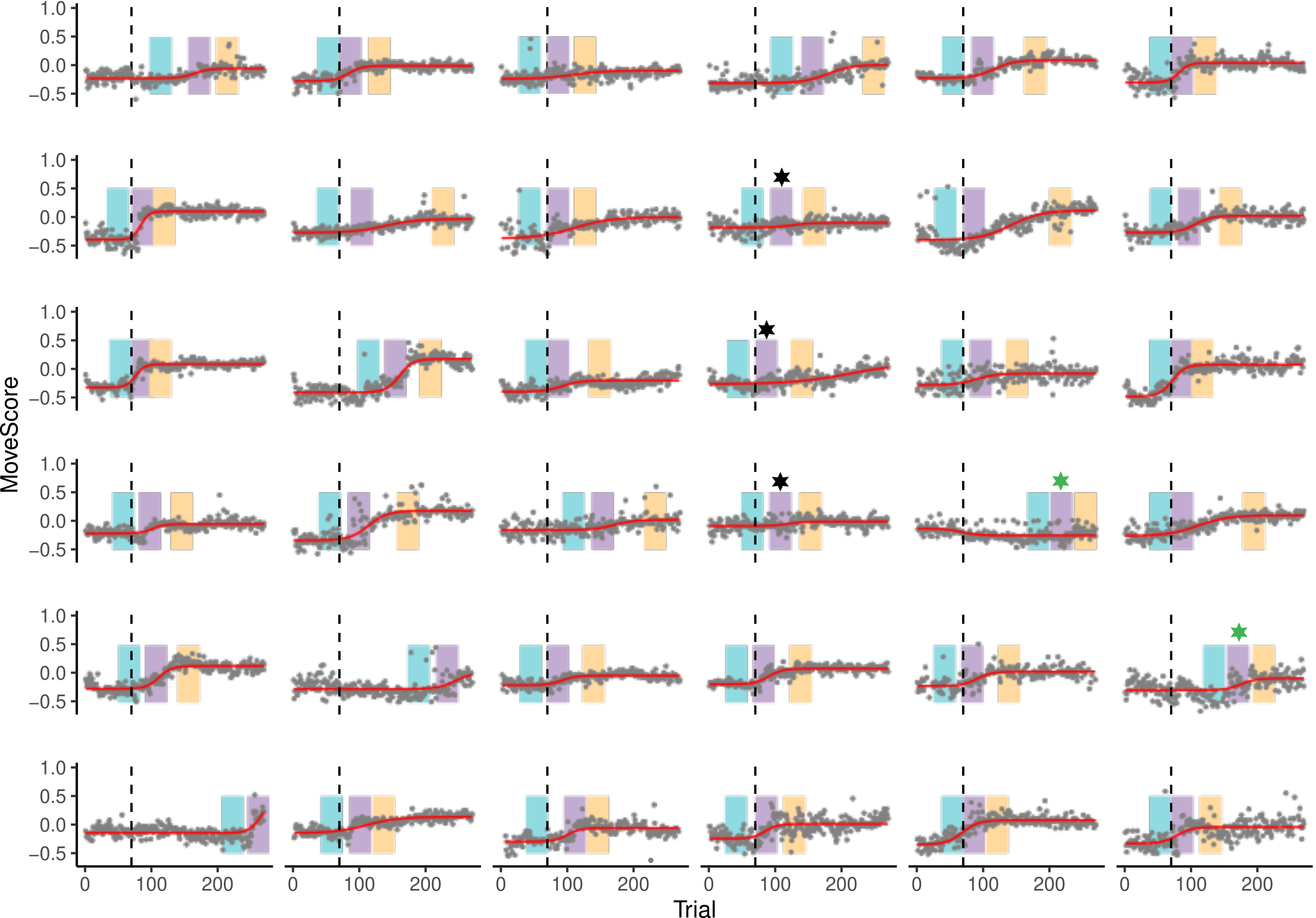

Supplement: S4 Fig — (TIF) [file pbio.3002934.s004.tif]

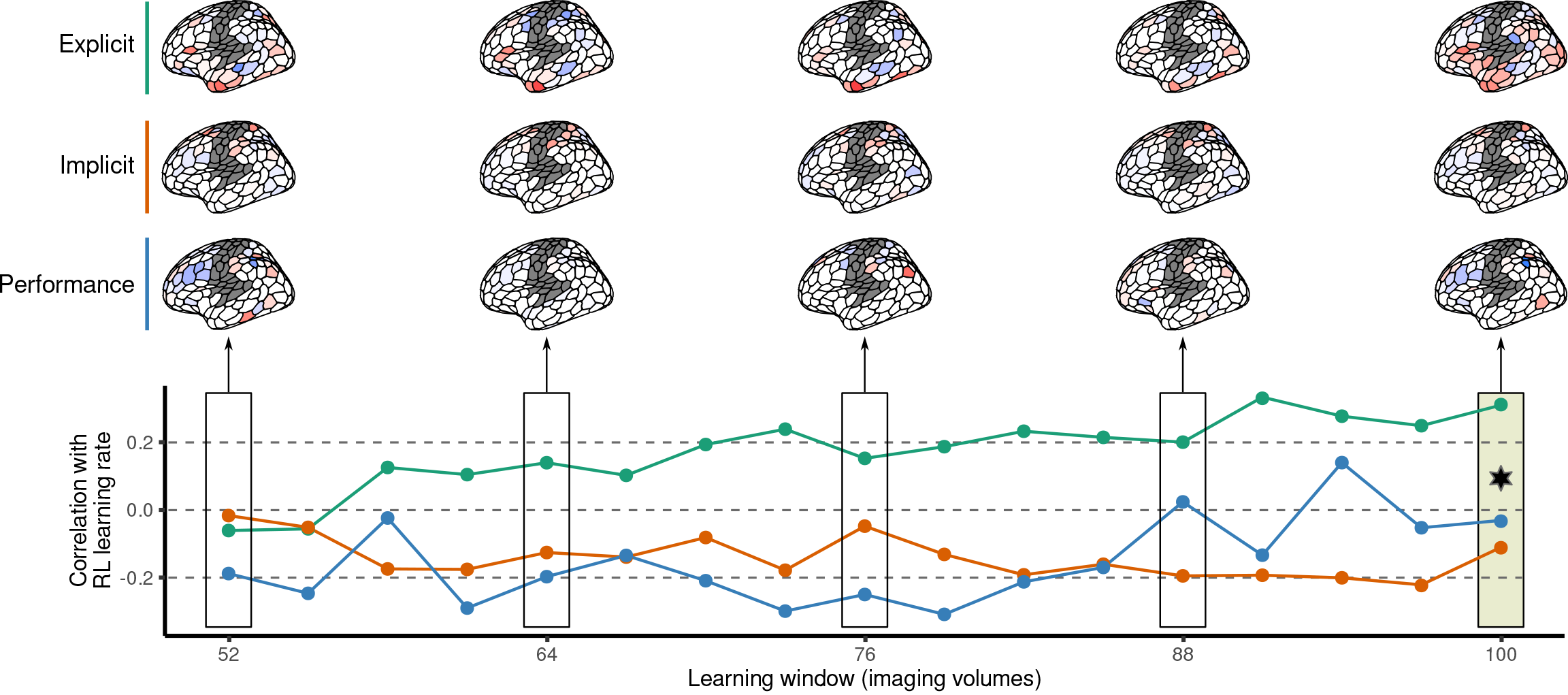

Supplement: S5 Fig — (TIF) [file pbio.3002934.s005.tif]

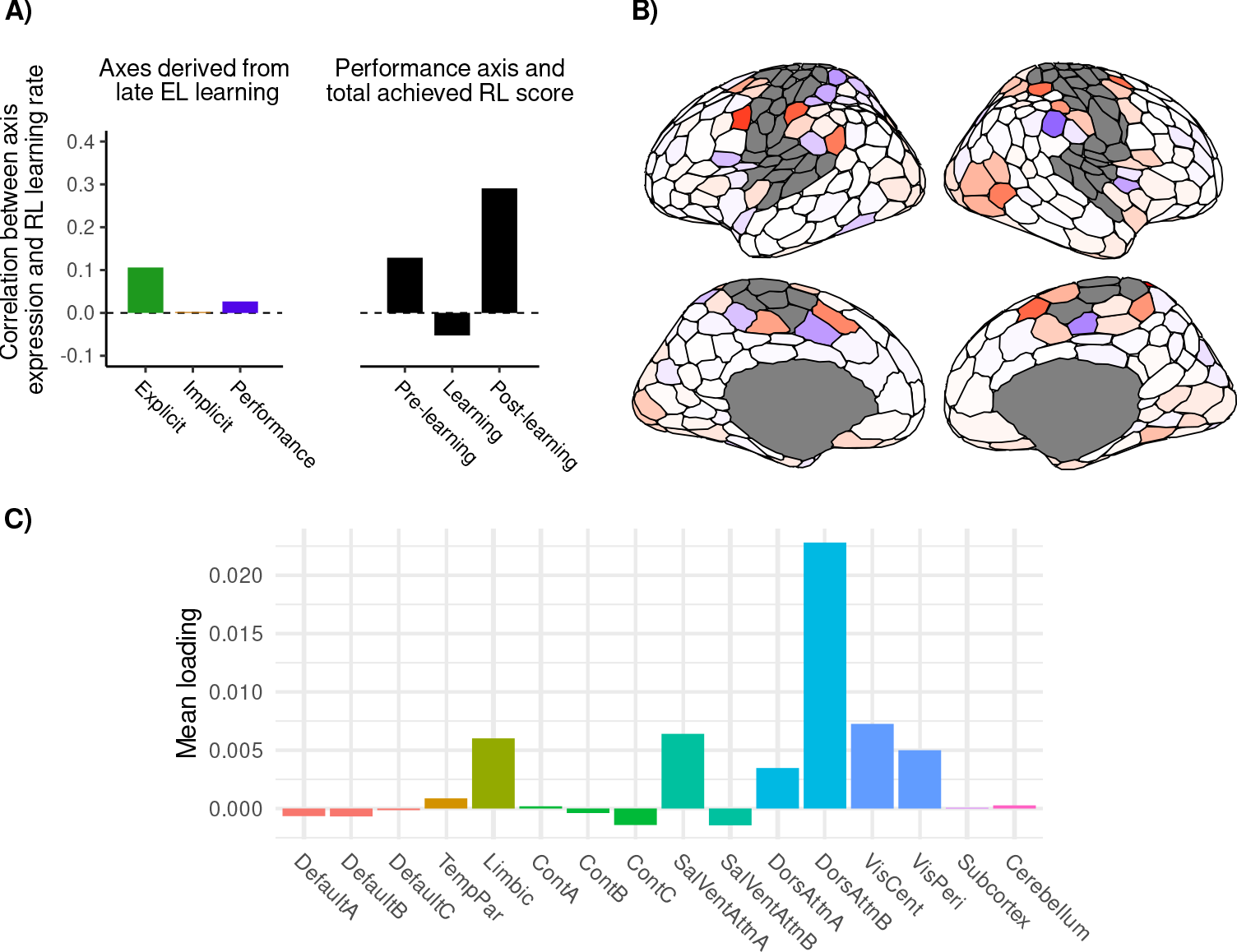

Supplement: S6 Fig — (TIF) [file pbio.3002934.s006.tif]
