# Supplementary material for: Distinct patterns of connectivity with the motor cortex reflect different components of sensorimotor learning
Source: PLoS Biol. 2024 Dec 3;22(12):e3002934. doi: 10.1371/journal.pbio.3002934 (PMC11644839; doi:10.1371/journal.pbio.3002934)
Supplement: Attachment — Submitted filename: Reply2reviewers_PlosBilogy_June072024.pdf [file pbio.3002934.s007.pdf]

Summary of changes in Response to Reviewers:

We thank the reviewers for their time in reviewing our manuscript and for their helpful comments and suggestions. As we hope the reviewers will note in our response below, we have done a considerable amount of work to constructively address all the comments and suggestions provided by the 3 reviewers. In addition to the pages that follow, we have made considerable changes to the manuscript and supplementary material to incorporate and address each of the concerns. We believe these changes have strengthened and improved the overall impact of the paper.

To avoid unnecessary repetition in our responses to the 3 reviewers below, below we briefly summarize, in point form, the major revisions made to the paper to constructively address the major comments from the reviewers. For details concerning other revisions, we kindly ask the reviewers to see our reply to their specific comments in the sections that follow.

Major revision #1: Validation of subjects' explicit reports through a separate behavioral study

Several reviewers expressed some skepticism about the validity of us having obtained subjects' explicit reports (i.e., their re-aiming strategies) after learning had already taken place in the VMR task. As we noted in our manuscript — and as a few of the reviewers seemed to have appreciated — we included the reporting period at the end of learning so as to not bias subjects' learning performance. Indeed, in our previous work we have shown that the reporting procedure itself increases the likelihood that subjects will adopt an explicit aiming strategy (see De Brouwer et al., 2018), and this would have been a major potential issue for us given that our fMRI study is strongly focused on individual differences. Our decision to have the reporting procedure performed at the end of learning was based on our anecdotal observation across our own previous studies in the lab that the explicit reporting on late trials is a reliable indicator of subjects' reporting on early trials. However, we now realize based on the reviewers' comments that we did not explicitly state this assumption in our paper, nor did we provide any concrete behavioral evidence to support this contention.

To rectify this omission, we have now performed (and included) an additional behavioral experiment in the supplement (in 14 participants), in which we elicited reports intermittently throughout the VMR task (as in our previous work, De Brouwer et al., 2018). As we show in this new experiment, subjects' reported re-aiming during the early learning window correlated very strongly (r=0.77) with their explicit reports elicited at the end of the task. Further, this study also shows that subjects tended to develop explicit awareness of the rotation very abruptly during the early stages of learning, and that their degree of explicit knowledge subsequently remained quite constant throughout the entire learning period. We believe that the inclusion of these new behavioral results in our paper help to validate our experimental approach, and confirm that: (1) the formation of an explicit strategy generally occurs during early learning (if at all), and (2) late reports are a reliable measure of the total degree of explicit re-aiming developed during the early learning period. We expect that the inclusion of this new data helps to bolster our original interpretation of the results.

Major revision # 2: Better discussion of brain networks and a more formalized interpretation of the learning axes

As one reviewer pointed out, our discussion of the brain networks used in our analysis, and of the neural axes themselves, was perhaps a bit esoteric for readers less familiar with the fMRI network neuroscience literature. Moreover, we appreciate that we may not have done a great job of providing the reader with an overarching framework for how we think about the current findings in

relation to the existing literature. To rectify this, we have now included a new figure in our Discussion section, as well as several new Discussion paragraphs, that helps to contextualize our findings with respect to known properties of different brain networks. Finally, we have also more clearly explained both the rationale behind our decision to control for performance-related factors in the construction of our learning-related axes, as well as our interpretation of the performance axis.

Inclusion of several new fMRI control analyses
At the request of a few of the reviewers, we have now performed and included several new control analyses that clearly demonstrate the selectivity of the effects we reported in our original paper.

For instance, we now show that:

(1) an 'Explicit' neural axis derived during late VMR learning (after the development of an explicit strategy has already taken place) fails to predict performance in the reward-based motor task (addressing a suggestion from reviewer #3).

(2) the expression of the Explicit axis during *either* the pre-learning or post-learning periods of the reward-based motor task does not predict performance (addressing the concerns of reviewer #2).

Together, these two new control analyses, in combination with our findings that neither connectivity with the tongue or ipsilateral hand motor areas can predict performance in the reward-based motor task, strongly supports our contention that our Explicit axis reflects processes involved specifically in the explicit *learning* process itself.

In addition, as reviewer #2 pointed out, the failure of the cerebellum to appear prominently in our implicit learning axis contradicts the well understood role of the cerebellum in implicit, error-based adaptation. In our Discussion, we speculated that this null finding was due to relatively weaker cortico-cerebellar functional connectivity, which caused cortico-cortico connections to be preferentially selected for by our model. We recognize, however, that this methodological explanation may be unsatisfying. To rectify this, we have now included an additional control analysis (reported in the supplement) focusing specifically on cerebellar connectivity with the motor cortex. This analysis confirms that cerebellar functional connectivity behaves sensibly during learning — e.g. (1) connectivity with motor cortex during task performance is increased primarily in ipsilateral cerebellar motor regions, and (2) the correlation between functional connectivity and implicit learning is localized primarily to motor regions of the cerebellum.

Given these substantial changes noted above, as well as several other changes noted below, we hope that you now find the manuscript suitable for publication.

Throughout our response, we will use the following format:

(1) black text in boldface is used when we directly quote the reviewers;
(2) black plain text denotes excerpts from the original manuscript;
(3) blue text is our response; and
(4) red text is used when quoting from the revised manuscript.

Reviewer remarks:

Reviewer #1:

Summary: Areshenkoff et al. examined how functional connectivity with motor cortex varies for explicit, implicit, and performance measures during visuomotor adaptation. Their findings indicate that explicit learning is primarily linked to the default network, implicit learning is linked to the dorsal attention network, and performance is linked to the control network. Together, this research deepens our understanding of how neural networks might be involved in motor adaptation.

Evaluation: This valuable study by Areshenkoff et al. reveals the functional neural networks correlated with different processes supporting motor learning. Although the authors offer fresh connectivity perspective on this fundamental question, my enthusiasm is tempered by concerns about the experimental design and imaging analyses. This work will be of considerable interest to researchers working at the intersection of motor learning and decision making.

Author Reply:
We thank the reviewer for their helpful comments and suggestions, and address these and other points in detail below.

Recommendations:

1. The authors did not measure explicit learning during the early phase, making the interpretation of functional connectivity during this early period challenging. Specifically, the degree of explicit aiming can significantly differ between early and late learning stages (e.g., from 30 degrees in early learning to 15 degrees in late learning) as seen in previous studies (Tsay et al. 2023; Taylor, Krakauer, and Ivry 2014). For example, many participants do not show signs of explicit re-aiming in late learning (Figure 1), yet it may be possible that they have engaged in considerable explicit aiming during the early learning phase (the period used for functional connectivity analyses).

Author reply:
We appreciate this thoughtful comment from the reviewer (which was also echoed by the other reviewers). As noted in our preamble above, we have now performed a separate behavioral study in 14 participants to investigate the relationship between subjects' magnitude of explicit re-aiming during the early versus late learning imaging windows. [Note that this behavioral study was identical in design and trial number etc. to the one we performed in the MRI scanner]. As demonstrated in this behavioral study, we observed a strong positive relationship (r=0.77) between subjects' explicit reports during these two time periods (see figure below). In addition, as can be observed in the single subject data, we found that, of the subjects who reporting using an explicit re-aiming strategy, this strategy (1) was formed quite early during the learning period (i.e., during the early learning window) and (2) was quite stable across the full learning period (i.e., we did not find evidence that subjects' explicit aiming significantly differed between the early and late learning periods). With respect to the latter point (and the reviewer's comment above), we did not observe any cases in which subjects engaged in an explicit re-aiming strategy during the early learning phase that was not also present (and observable through their reports) during the late learning phase.

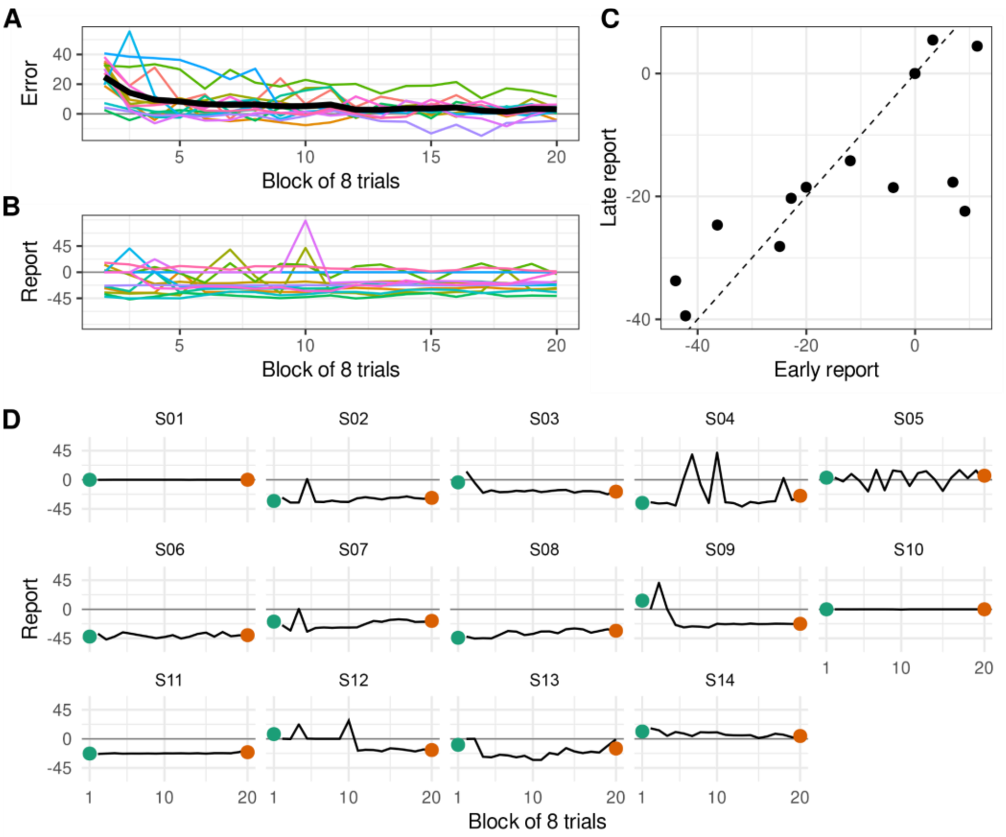

We have now included these findings as a paragraph in our Results section (see below), and as a figure in the supplement. Specifically, in our Results section we now write the following:

“Our decision to elicit subjects’ reports at the end of the learning block was based on evidence that report trials may make subjects aware of the visuomotor rotation and predispose them to use an explicit strategy during the task (de Brouwer et al., 2018; Maresch et al., 2021). Nevertheless, group-level behavioural studies suggest that subjects’ explicit re-aiming strategies may evolve during learning the process (e.g., have a larger magnitude during early than late learning; Taylor et al., 2010, Taylor et al., 2014). As such, reports collected at the end of the task may not directly reflect the re-aiming strategy subjects used during the early learning process itself. To address this possible concern, we conducted a separate behavioral experiment outside of the MRI scanner (N=14), in which report trials were interspersed continuously throughout the learning block (see Supplemental Figure 1). Importantly, we found that subjects’ explicit reports collected in the final trials of the learning block were highly correlated with reports collected during the early learning block ($r = 0.77$, $t_{12} = 4.22$, $p = 0.0012$). Further, we found that subjects’ typically developed explicit awareness of the rotation early in the learning block, and that their subsequent explicit reports were highly stable throughout the VMR task. Taken together, these findings suggest that the late reports collected during our MRI testing session serve as a valid proxy for the explicit strategy developed during this early learning period.”

The authors could consider an alternative proxy for explicit re-aiming: the difference in reaction time between the early and baseline periods. A larger change in reaction times

could indicate more extensive use of explicit strategies (Fernandez-Ruiz et al. 2011). Therefore, one could ask how this change in reaction time correlates with the variations across networks, and whether an analysis focused on the RT measure jives with those derived from late aim reports.

Author reply:
This is an interesting suggestion from the reviewer. Indeed, consistent with the reviewers' expectations, and with the previous literature (e.g., Fernandez-Ruiz et al., 2011), when we examined the relationship between subjects' early learning and late learning RTs with their aiming reports, we observed a strong positive correlation in both cases. We have now included these nice results in our Behavioral data figure associated with the VMR task (see Fig. 1D, see below). However, because RTs are also known to correlate with several other cognitive variables (e.g., attentional state, task difficulty, etc.), we are not completely confident about solely using RTs as a direct proxy for explicit strategy use. Instead, we hope that, with the inclusion of the new behavioral study above, we have constructively addressed any potential concerns about the validity of our explicit reports, and thus the interpretation of our results.

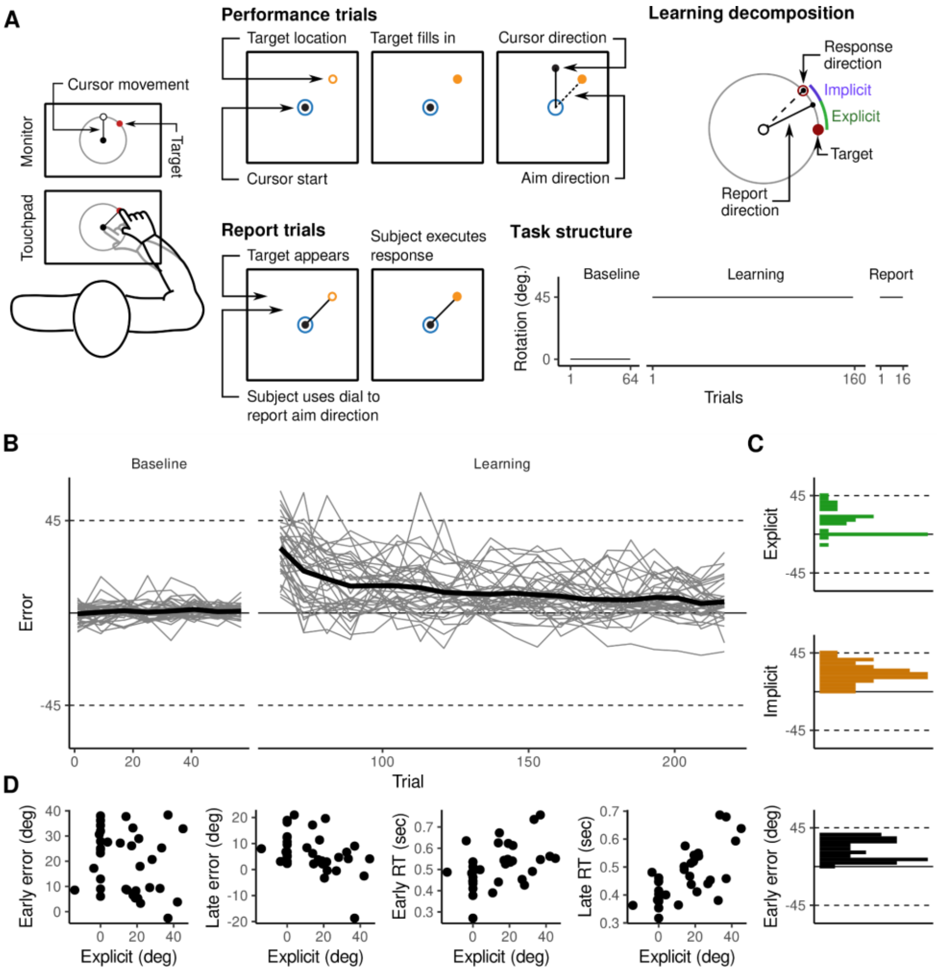

2. Related to the comment above, I recommend that the authors provide a more detailed characterization of the learning curves and kinematic measures (e.g., reaction time and

movement time) in both experiments, so readers can better appreciate the behavioural dynamics that unfold throughout the experiment.

Author Reply:
Thanks for this comment. We have now done a much more thorough behavioral analysis for both the VMR and reward-based motor learning datasets in the manuscript (including analyses of RTs and MTs). Please see our new figures (Figs. 1 and 5) for these more detailed behavioral quantifications.

3. The authors identified different networks associated with overall performance, which can be dissociated from those related to implicit/explicit processes (Figure 2). However, I am puzzled by the assumptions and implementation of this analysis. Assuming that total performance is the sum of explicit and implicit components, how did the authors derive a connectivity measure associated with performance that is distinctly different (orthogonal) from those of other processes (i.e., why is the control A network not involved in either implicit or explicit, when they both contribute to total performance)?

Author Reply:
We appreciate the opportunity to clarify this, and have now done a more thorough job of explaining the exact construction of these measures, and of explaining their interpretations. In particular, we now make it clearer that our intention was to separate patterns of connectivity associated relatively "purely" with implicit or explicit processes, from effects related to general task performance. For example, regions of the control A network (e.g. dlPFC) are involved in the selection of spatial targets for action, which is likely to be necessary for task performance regardless whether learning occurs primarily through implicit or explicit learning mechanisms. Our goal was to partial these more "general task-based" processes out of our explicit and implicit axes, and group them under a general 'Performance' axis.

In the Results section of our revised manuscript we now discuss the implementation of these analyses in the following text:

"Note that, because both implicit and explicit learning result in better performance (less error), the neural correlates of these measures may also reflect effects due to the experience of errors. For example, systems involved in the goal-directed deployment of spatial attention may be necessary for successful task performance regardless of the specific mechanisms by which a given subject learns, and so may be recruited by both implicit and explicit learners. Thus, in order to derive relatively "pure" measures of these two learning processes, we controlled for subjects' performance by using the mean error during the early learning epoch as a covariate in our model. Specifically, we separately regressed each of our explicit and implicit learning measures, and the functional connectivity of each ROI with motor cortex, onto subjects' early errors, and used each of the residuals for further analysis. Separately, in order to derive neural activity patterns linked to learning performance per se, rather than any particular learning process, we also derived patterns of functional connectivity associated with subjects' visuomotor errors. We interpret this 'Performance' axis as reflecting processes related to general error-detection, or to general task-based processes which may be common to both forms of learning".

We hope that these changes to the text now make it much clearer our intentions and procedures for the analysis.

4. Related to above, while I understand why implicit networks might not generalize to the learning rate in the reward-based task (assumed to be explicit), it is not clear why the

networks related to performance are not predictive of learning rate – doesn't the reward task also involve changes in performance?

Author Reply:
Good question. There are several key differences between the error-based and reward-based learning tasks which we believe may drive this lack of relationship. The most important of which is the fact that learning in the reward-based task is overwhelmingly explicit, and so behavioral changes during the clearly defined learning periods are likely to rely largely on brain systems involved in explicit learning processes (i.e. those comprising our explicit axis). Another possibility is that some of the effects comprising our performance axis could relate to general spatial processes necessary for performance in the VMR task (e.g. identifying spatial targets for action), which are largely irrelevant to the reward-based task. We do note – and have now included in our supplemental control analyses (Supplementary Fig. 5) – that we observed a moderate (though non-significant) correlation between expression of the performance axis during the post-learning period and subjects' attained reward-based score (the upper asymptote of the sigmoid fit). This may be consistent with our speculation that the performance axis may include, partly, processes related to general performance monitoring.

In any case, based on the reviewer's comments, we have now made major changes to the Discussion section to address these points. We now write the following:

"A second key feature of our results is the distinction between two separate patterns of functional connectivity during sensorimotor learning: one linked to explicit learning and the other linked to visuomotor performance. In prior work, explicit learning has been suggested to involve higher-order control regions in the prefrontal cortex, as well as processes like working memory and attention (Areshenkoff et al., 2022; McDougle et al., 2016). Yet, the specific influence of these control signals on motor system parameters has been unclear. For instance, it remains to be formalized whether these control signals dictate the selection of a new policy (i.e. a decision to aim somewhere other than directly at the target), the selection of an alternative forward model, or the weighting of specific sensory inputs based on task demands. Indeed, regions of the prefrontal cortex have been implicated in exactly these types of processes (e.g. Bédard and Sanes, 2014) and associated with performance during visuomotor adaptation (Taylor et al., 2010; Taylor and Ivry, 2012; Areshenkoff et al., 2022; McDougle et al., 2016). However, the extent to which these Control networks specifically contribute to the learning of an explicit strategy per se – versus processes which may support task performance more generally (e.g., goal-directed spatial attention), and thus lead to better performance – has not been systematically studied in previous work. Indeed, Control network areas like the DLPFC have been implicated in processes essential for basic task performance – such as the selection of spatial targets for action and with performance monitoring (Iba and Sawaguchi, 2003; Mitchell et al., 2008; Holroyd and Yeung, 2012) – in addition to any specific role in explicit learning. Our current results suggest that functional connectivity with the DLPFC (Control-A subnetwork) is not associated with explicit learning specifically, but rather with processes that assist in task performance (e.g., spatial attention, error detection). The fact that expression of this axis was not correlated with learning rate in the separate reward-based motor task may thus reflect the fact that this axis includes (in part) patterns of connectivity associated with the unique demands of the VMR task (e.g. spatial target processes), which are not relevant to performance in the reward-based task, and/or with effects related to the monitoring of performance (e.g., detection of directional errors), rather than processes contributing to the formation of a strategy."

5. A recent perceptual/proprioceptive re-alignment hypothesis posits that implicit adaptation is driven by a perceptual error (a mismatch between perceived movement

position and the target; (Tsay et al. 2022; Zhang et al. 2024)). Implicit adaptation therefore ceases when the perceived and desired position of the hand are aligned. Importantly, the perceived movement is derived based on a multisensory estimate combined with an efference copy, which seems to jive nicely with the implicit networks identified (parietal/premotor: multisensory regions (Fang et al. 2019; Chancel, Iriye, and Ehrsson 2022); anterior cerebellum: efference copy (Bhanpuri, Okamura, and Bastian 2013)).

Author Reply:
Thanks for reminding us about this important work. We agree that it jives very nicely with findings in the dorsal attention network (DAN), and thus merits a brief discussion in our paper. To this end, in our Discussion section, we now write the following:

"A key finding from our study is to underscore the important role of the superior parietal and premotor areas within the dorsal attention network (DAN) in implicit adaptation (see Figure 7; bottom). This learning process is frequently understood through the lens of optimal feedback control (Scott, 2004), where the observed response results from a policy dictating the motor control signal generated by primary motor structures. Successful execution of this policy requires an internal model to generate accurate predictions about the sensory consequences of a motor command. Recent perspectives suggest that this model is updated based on perceptual errors, reflecting a mismatch between the target and the perceived movement position, which is derived from a multisensory estimate combined with a motor efference copy (Tsay et al., 2022). Previous work has implicated regions of the parietal and premotor cortex, as well as the anterior cerebellum, in these processes (Fang et al., 2019; Chancel et al., 2022; Bhanpuri et al., 2013; Scott, 2012). Note that although our findings identified these same cortical regions (in the DAN), we found that the cerebellum loaded relatively weakly onto our Implicit neural axis. However, we should note that its anterior motor regions – particularly in the right (ipsilateral) cerebellum – were aligned to our Implicit neural axis, consistent with its known role in computing the sensory prediction errors that drive implicit motor adaptation (Smith et al., 2006; Tseng et al., 2007; Wolpert et al., 2011; Manto et al., 2012; Tanaka et al., 2020). "

Thanks again for your comments and feedback to help improve the manuscript.

Reviewer #2: The goal of this paper is to characterize the underlying neural systems that mediate the implicit and explicit components of visuomotor adaptation, as well as error processing during this task. To do so the authors use functional MRI and study whole-brain patterns of functional connectivity with motor cortex. They identify three distinct axes of whole-brain connectivity with the motor cortex that emerge during early learning: implicit learning is associated with connectivity in superior parietal and premotor cortex; explicit learning is associated with regions of the default mode network; and errors (visuomotor performance) are associated with connectivity in regions of the frontoparietal control network. The authors provide confirmation for the contribution of the DMN axis in a reinforcement learning task that specifically engages explicit mechanisms.

This paper reports on an extensive series of analyses of changes in connectivity with M1 during visuomotor adaptation, as well as in a reinforcement learning task. The framing of the work is good, and it is very fair to say that the characterization of the neural underpinnings of the implicit and explicit components is an important question. Both from a behavioural and neuroimaging standpoint, the work is conducted with rigor and according to high standards. There are however, a lot of seemingly arbitrary choices regarding data reduction, and a lot of data manipulation that complexify the story and question the robustness of the findings. The networks identified are somewhat unexpected (no cerebellum for implicit, DMN for explicit), and leave an aftertaste of whether these complex and quite opaque analyses were bound to generate significant results, whatever the regions or networks. The paper would benefit from confirmatory findings for regions that are expected to show an effect, or a more formal null result type of analysis that would confirm that significant networks don't come about spuriously.

Author Reply:
We thank the reviewer for their comments and suggestions. While it is true that our regression-based analysis of the VMR task data was bound to generate results of *some* kind (that is, we could always have derived *some* axes, even if they were only noise), the fundamental test of the authenticity of these axes is in their ability to generate across-task predictions, which was the focus of our second analysis. Moreover, in the case of the Implicit axis, which (as expected) did not predict performance in a separate task, the loadings themselves were entirely consistent with the known circuitry underlying implicit adaptation in the VMR task. Likewise, in the case of the explicit axis, the successful across-task prediction is clear evidence that the axis itself was not merely noise. Moreover, we validated this latter finding using several different controls that go well beyond current standards in the neuroimaging literature (e.g., that across-task prediction did not occur during either the pre-learning or post-learning periods in the reward-based task, nor when using connectivity with the tongue motor brain areas, etc.). Further, as we now better explain in the Discussion, while the involvement of the DMN may be unexpected to researchers within the sensorimotor learning community, it is entirely consistent with an emerging consensus on the role of this system in strategic learning more generally (as well as with several seemingly forgotten results in the early motor neuroimaging literature).

We address these and other points in detail below.

Major comments

One cannot disagree with the regions that were identified with the implicit component. Parietal and premotor cortex must be at play (they could have been at play for the error axis too). However, the fact that cerebellum did not come out is perplexing.

I know the authors address this in the conclusion, and leave to others the "construction of models based solely on cortico-extracortical connectivity to more precisely identify the role of extracortical regions in different learning processes". Still, the reader might have expected that the authors perform such an analysis here, to increase credibility of the analysis pipeline.

Author Reply:
We appreciate the reviewer's comments re: our null findings in the cerebellum. We really do think that our lack of a cerebellar effect stems from the connectivity between motor cortex and the cerebellum being relatively weaker (by comparison to intra-cortical connectivity), and thus leading to a down-weighting of those connections in the group-penalized regression model.

Nevertheless, to assuage some of the reviewers' concerns re: our data quality and interpretation of the results, we have taken their advice and performed a separate functional connectivity analysis based only on connections between the cerebellum and motor cortex. We have included the results of this analysis as a new supplemental figure, and also include them below for the reviewer.

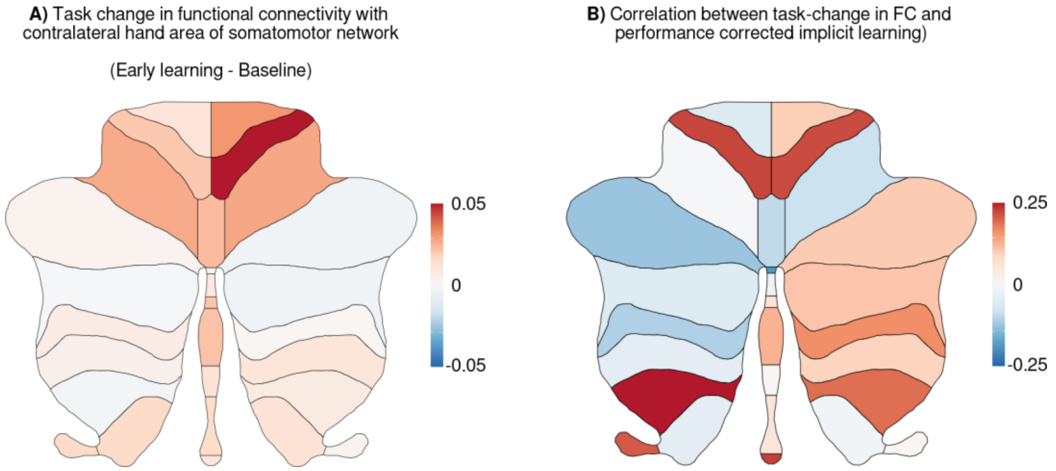

As can be seen in the figure above, when we examined the regions of the cerebellum that selectively changed their functional connectivity (FC) with the contralateral hand area of motor cortex during learning trials (compared to baseline), we mainly observed increased FC in the anterior motor areas of the cerebellum (see red areas in panel A), and particularly in the right cerebellum, consistent with its known connectivity with left motor cortex. Moreover, when we examined correlations between this increase in FC and subjects' performance-corrected implicit learning behavioral measure, we again found positive correlations in the anterior motor cerebellum — and particularly in the right cerebellum. While it is important to note that none of these correlation-related effects were statistically significant, they nevertheless conform with general expectations and hypotheses that could be derived from the previous literature. We hope that the inclusion of these results in the supplemental material helps add credibility to our overall results.

Moreover, from reading the intro one would have expected that the authors also perform a corollary of the RL task to test the generalization for the implicit component, using a ramp protocol for instance, in which the contribution of the implicit is maximized and that of the explicit nulled. But I understand that at this point this may not be feasible.

Author reply:
This is an interesting idea, but probably one that would not translate well to the current tasks and conditions. First, to obtain implicit learning during the motor RL task, one would likely require use of a different kind of task. As we noted in our paper, motor RL tasks tend to elicit very little implicit RL (for some examples, see de Brouwer et al., 2022; Dam et al., 2013, Holland et al., 2018; Codol et al., 2018). Indeed, it was this very feature of motor RL that prompted us to use it as an independent task to test the idea that the Explicit learning axis is generalizable across tasks. Second, the learning processes that support implicit sensorimotor adaptation (sensory prediction errors) versus implicit motor RL (reward prediction errors) are already hypothesized to rely on fundamentally different neural pathways (e.g., Doya 1999, 2000). As such, we would have no expectation that the neural basis of implicit adaptation should generalize across to implicit reward-based motor learning (and vice versa). It is true that we could have run this separate study and analysis, but we assume that a resulting null effect (as would be predicted) would not be entirely convincing. We tried to capture these important points in our Results section when motivating the second reward-based motor learning study:

"Implicit adaptation in the VMR task is thought to primarily reflect the updating, through sensory prediction errors, of an internal forward model that predicts the sensory consequences of a motor command (Wolpert, 1997; Mazzoni and Krakauer, 2006)). In other forms of motor learning, where subjects are denied this sensory error feedback, this same kind of updating is impossible, and so learning in such cases must rely on alternative neural systems and pathways (Doya, 2000; Wolpert et al., 2011). By contrast, explicit learning has been linked to various executive functions (Anguera et al., 2010; Seidler et al., 2012; Drummond et al., 2015; Taylor and Ivry, 2014), and the DMN – which features prominently in our Explicit neural axis – is suggested to specialize in cognitive computations that are independent of specific perceptual inputs (Smallwood et al., 2021). We thus reasoned that the Explicit neural axis might generalize across motor learning tasks that provide different forms of sensory feedback, whereas the implicit neural axis would not."

In addition, we also tried to reiterate these same points in the Discussion:

"A final unique feature of the current study was in exploring the extent to which the neural axes identified during sensorimotor adaptation generalized across other forms of motor learning. Although few neural studies have examined the use of reinforcement mechanisms during motor learning, there is considerable behavioural evidence indicating that explicit processes are particularly crucial in tasks that deny the kinds of sensory feedback that drive error-based adaptation; such as in our reward-based motor task, or the tasks of Holland et al. (2018); de Brouwer et al. (2022); Dam et al. (2013); Wu et al. (2014). In these types of reward-based motor learning tasks, the agent is provided no feedback indicating precisely how, or to what extent, the executed versus optimal movements differ (unlike during error-based learning), and so they must perform some kind of credit assignment in order to decide which feature of the movement to adjust on subsequent trials (Sutton and Barto, 2018). In these contexts, subjects will often develop an explicit understanding of the task structure, with their motor responses demonstrating a clear prioritization of the features relevant to performance (Dam et al., 2013; de Brouwer et al., 2022). Consistent with this, the majority of subjects in our reward-based motor task exhibited well defined learning periods, in which subjects began to more rapidly converge upon the correct solution. These more rapid shifts in behaviour have been studied in the context of one-shot learning, where research has implicated regions of the DMN (Lee et al., 2015) in facilitating rapid learning through an explicit record of past action-outcome associations (Doll et al., 2015). As noted above, these brain regions featured prominently in our Explicit neural axis, the expression of which predicted subject performance in the reward-based motor task."

We hope that these sections make it clear our expectation that the only shared component between the VMR and reward-based motor tasks should be the explicit learning process.

I appreciate that the authors performed the reinforcement task to bolster the finding that DMN might mediate the explicit component. However, as far as I can understand this bears on a correlation that stands at 0.049. This is a little worrisome given the tricky nature of this task, as attested by the heterogeneous pattern of adaptation across participants, and some of the arbitrary decisions regarding the analysis pipeline. For instance, what is the justification for choosing these specific 100 volumes going into the fMRI analysis? The aha moment is highly debatable, especially for those participants who never really learned (the 2 identified manually).

Is it fair to say that cognitive deliberation in search of a solution really only happens in the 100 volumes following the 5% of the slope? One could be quite engaged much before, when errors are large.

My point is that any difference in any step of this long chain of decisions might have altered the results. I think the authors need to provide additional tests of robustness for this analysis in order to bolster the utility of this task.

Author reply:
We appreciate the reviewers' skepticism and their request for additional tests of robustness. As we noted in our preamble at the beginning of this document (and discuss more below), we have now performed several such tests for our revised manuscript, but just thought we should clarify a few points first:

(1) Our analysis on 100-volume imaging windows was based on our recent work in this area (Areshenkoff et al., 2022 Elife), which also used the exact same time-window lengths. Thus, we didn't cherry-pick our window lengths, if that is any concern.

(2) While we agree that one could quibble about our usage of the term 'Aha moment' (which may evoke notions of 'single-trial learning'), we do not think it is debatable that the majority of subjects exhibited clearly defined learning periods. Indeed, it was the obviousness of these learning periods — which were book-ended by periods of flat performance — that was our very motivation for using sigmoid fits to define these periods in a purely data-driven fashion. It is true that this sigmoid-function fitting procedure failed in a few subjects (requiring our manual intervention), but this is only because those subjects exhibited flat or completely non-existent learning — i.e., the very cases that help prove the rule. Indeed, for these subjects, regardless of the specific learning period chosen for them, the end result of the fitting procedure was a very low learning rate (miniscule *k* parameter), which is entirely sensible given their lack of learning.

With respect to the reviewer's critique about the temporal selectivity of our findings (i.e., that cognitive deliberation somehow only happens within the 100 volumes following 5% of the slope), we do appreciate the point they are making. The 5% criteria was merely a data-driven threshold applied to the learning data to make the analysis consistent and tractable across subjects, and we don't mean to convey the idea that subjects are not involved in cognitive deliberation prior to this time period. However, what now seems clear — especially after having performed a new control analysis prompted by this reviewer (see below, and thank you for suggesting it!) — is that any expression of the Explicit neural axis prior to the learning period is not correlated with performance during the reward-based task.

Recall in our original submission that we had only performed a control analysis using the post-learning period of the reward-based motor task, which resulted in a weak, non-significant correlation (r=-0.18, p=0.32). However, prompted by this reviewer's comment, we have now also gone back and performed a control analysis also using the pre-learning period, as defined from our sigmoid fits. Consistent with our findings being selective to the learning period, we again found a weak, non-significant correlation between the expression of the Explicit axis and reward task performance (r=-0.06, p=0.74) when using only the pre-learning connectivity. Together, these results suggest that there is something unique about the Explicit neural axis being expressed during the learning period which leads to faster learning (and which is not the case during both the pre-learning and post-learning periods). When combined with several of our control analyses (noted in our preamble), this result strongly suggests that the neural processes supporting explicit *learning* (i.e., formation of the learning strategy) are shared across the VMR and reward-based tasks, and that these neural processes are behaviourally relevant (i.e., we only find them when examining correlations with the contralateral hand area of motor cortex).

We now highlight these new pre-learning control findings in our Results with the following text:

"Moreover, as a further control, we also observed no correlation between the expression of the Explicit axis and performance in the reward learning task when we separately examined both the pre-learning ($r = -0.06$, $t34 = -0.34$, $p = 0.74$) and post-learning periods of the task ($r = -0.18$, $t34 = -1.01$, $p = 0.32$). Together, these results strongly suggest that the brain systems comprising our Explicit axis are recruited specifically during the learning period itself – when subjects are actively adjusting their behavior – rather than in the preceding and subsequent periods of relatively stable performance, respectively."

In addition, given the potential to misinterpret our intended meaning about the 'Aha moment', we have removed this term from our manuscript. Instead, we now just refer to these points in time as 'clearly defined learning periods'. Thanks.

On a side note, it also appears like a complex strategy to have resorted to relating learning-related increases in the expression of each neural axis to learning rates in the RL task. Couldn't the authors have used an approach more similar to the one used in the VMA task? Also, from a conceptual standpoint, it is counterintuitive that the errors are regressed out in the VMA task, but not in the RL task?

Author reply:
We would have to kindly disagree here. To us, this analysis approach was the most direct strategy to test the generalization of explicit learning across motor tasks — that is, through correlation testing we directly examined whether the Explicit neural axis from the VMR task is also expressed during the reward-based task, and if so, whether the magnitude of its expression relates to performance in the reward-based task. We can't actually think of another way in which this 'generalization' idea could be successfully tested.

Indeed, it is not obvious how one would have used a regression analysis approach in the reward-based task similar to the one we used in the VMR task, as in the reward-based task we did not separately measure explicit and implicit learning components. Thus, we would have only been able to generate a performance (score) neural axis in the reward-based task.

Finally, the point of regressing out errors from the VMR task is that it allows us to derive a relatively 'pure' measure of explicit learning (i.e., permitting us to examine neural activity patterns that are

correlated with formation of the learning strategy but not associated with visuomotor errors). Again, in the reward-based task we cannot regress subjects' score (our measure of their performance) against anything, as we do not obtain a separate measurement of subjects' explicit and implicit learning components in that reward-based task.

To summarize our basic argument: learning in the reward-based task is already known to be largely explicit, and appears to be confined to well-defined learning periods. To us, the most natural and straightforward way to examine whether the brain systems that support learning during these periods is indeed "task-general", is to derive a "pure" neural measure of explicit learning in a separate (VMR) task — which required controlling for effects related to the experience of errors (or other effects related to basic task performance) — and then ask whether the expression of this explicit neural component correlates with performance during the learning period in the reward-based task. This is exactly what we show in our paper.

Performance is very heterogeneous across participants in the RL task. Are these variations in performance related to the frontoparietal control network?

Author reply:
As we show in the paper, the variations in subject learning rate during the reward-based task — specifically during the learning period — were related to the expression of the Explicit neural axis, which has its strongest loadings on the DMN network (specifically the DMN-B network), but which also contains some smaller positive loadings from the Control C network (as we highlighted in our paper).

However, the reviewer might find it interesting that we also observed (shown the new supplemental control analyses) a moderate (albeit non-significant) positive correlation between the expression of the Performance axis (featuring regions of the Control network most prominently) with the overall score attained during the post-learning period (i.e., the upper asymptote of the sigmoid fit). Because this effect is non-significant, we are cautious about strongly interpreting this effect; however, we thought we would share that result as the reviewer might find it relevant (see figure below).

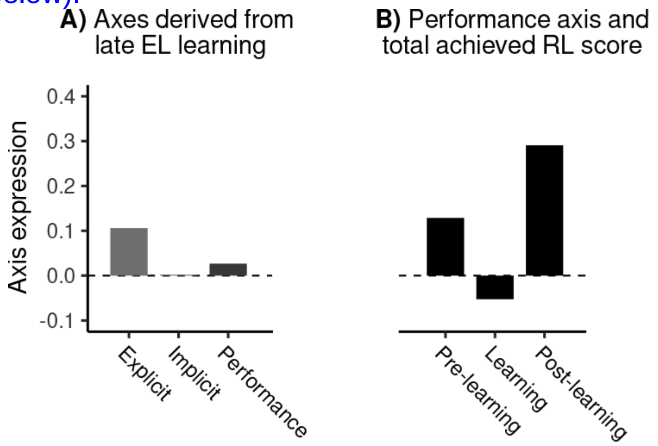

Only 8/16 report trials are being used given participants' confusion regarding the first 8 trials. This is worrisome and questions the validity of that estimate. This is critical since this measure is central to all subsequent analyses. Another source of concern is that it is not correlated with early learning performance, which would be expected (as the authors mention in the intro: "since explicit processes are often brought to bear at critical moments during learning, and can result in a rapid reduction in visuomotor errors [13]."). Could it

be because the report trials are only measured at the very end of the experiment, and thus poorly reflect aiming during the early trials? This appears like a limitation.

Author reply:
The reviewer makes two separate points here — one about using only a subset of the report trials, and the second about measuring report trials only at the very end of the experiment. We address these two points separately below.

First, with respect to the reviewer's comment about only using the last 8 out of the 16 report trials, we did this because several subjects expressed initial confusion about the report trials, resulting in a few invalid responses in the first bin of 8 report trials, before subsequently understanding the requirements of this phase of the study. In this regard, it is important for us to note that our subjects were not 'coached' in any way during the reporting trials. They were simply provided on-screen text instructions about how to complete this phase of the task, and had to interpret the requirements of the task for themselves [Note that we opted for this 'hands-off' approach given our concern that any verbal instructions from the experimenter might bias their behavior].

In any case, we have now included, in the supplemental material, a figure that provides a direct comparison between the explicit and implicit learning components estimated from the full 16 trials versus the 8 trials used in the analyses. As this figure shows (see below), there is a near perfect correlation between the learning components estimated using these two approaches (r=0.95 for explicit reports, and r=0.80 for implicit adaptation). However, as the second bin of 8 trials offers a 'cleaner' measure of subjects' explicit strategy (uncorrupted by some faulty trials), we have opted to use this data for our analyses.

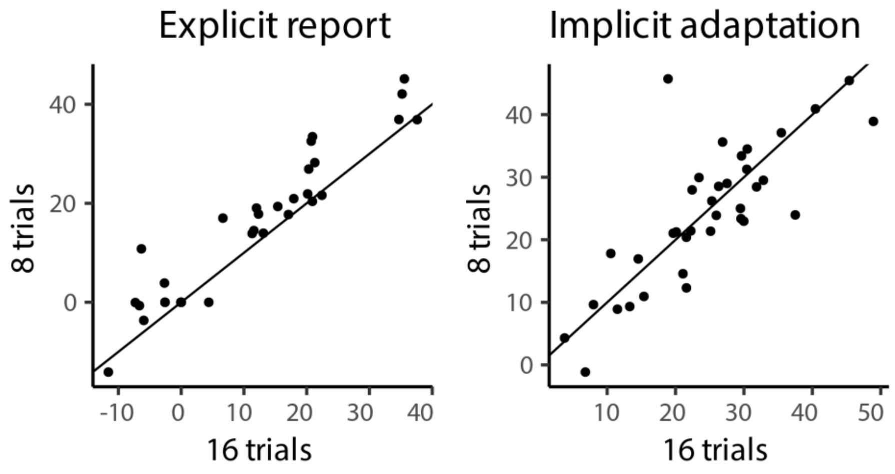

In addition, given that other readers may have similar questions about the nature of the reporting requirements, for the sake of transparency and replicability we have opted to include the exact text provided to participants (on the screen) at the onset of the reporting trials in our Methods section. In our Methods, we now write the following:

"At the beginning of the MRI testing session, but prior to the first scan being collected, participants re-acquainted themselves with the VMR task by performing 80 practice trials with veridical cursor feedback. Next, we collected an anatomical scan, followed by the baseline fMRI experimental run, wherein subjects performed 64 trials of the VMR task with veridical cursor feedback using their

right hand. Next, they performed the learning scan, wherein subjects performed 160 trials in which feedback of the cursor during the reach was rotated clockwise by 45◦. Following this fMRI experimental run, participants were asked to report their strategic aiming direction (over 16 trials). In these trials, a line between the start and target position appeared on the screen at the start of each trial. Participants were asked to use a separate MRI joystick (Current Designs, Inc.) positioned at their left hip to rotate the line to the direction that they would aim their finger movement in for the cursor to hit the target, and click the button on the joystick box when satisfied. Following the button click, the trial proceeded as a normal reach trial. Specifically, the following text was displayed on the screen to participants at the onset of the reporting trials: "Use the joystick to rotate the line to the direction that you will aim your finger in for the cursor to hit the target. If you think your finger should aim straight at the target, then leave the line at the target and click the button on the joystick. If you think your finger should aim somewhere else for the cursor to hit the target, rotate the line to your aiming direction, and then click the button. Next, hit the target with your right finger."

These 16 "report" trials were followed by 16 normal rotation trials. As several subjects expressed confusion about the nature of the report trials, often failing to adjust the line at all during the first few trials, we discarded the first 8 report trials for all subjects, and calculated the mean aim direction using the final 8 trials (note, however, that we observed very similar results whether estimates of the explicit and implicit components were based on these 8 versus all 16 report trials, see Supplemental Figure 2). Note that we had subjects perform these report trials following learning (as in de Brouwer et al., 2022) given our prior behavioural work showing that the inter-mixing of report trials during learning can lead to more participants adopting an explicit, re-aiming strategy (de Brouwer et al., 2018, 2022), thereby distorting participants' learning curves. Participants were not informed about nature or presence of the visuomotor rotation before or during the experiment, and received no "coaching" about how to specifically perform the reporting trials (we adopted this 'hands-off' approach given our concern that any experimenter instructions or invention might bias subjects' learning and reporting behaviour)."

Second, with respect to the reviewer's concern about the late reports possibly being a poor reflection of subjects' aiming during the early trials, as we noted in our preamble at the beginning of this reply document (and also in our reply to reviewer 1), we believe that we have now addressed this potential concern through a separate behavioral study. As noted, in this study we directly investigate the relationship between subjects' magnitude of explicit re-aiming during the early versus late learning time windows. Importantly, we observed a strong positive relationship between subjects' explicit reports during these two time periods (see figure below). In addition, as can be seen in the single subject data, of the subjects who reporting using an explicit re-aiming strategy, this strategy (1) was formed quite early during the learning period (i.e., during the early learning window) and (2) was quite stable across the full learning period (i.e., we did not find evidence that subjects' explicit aiming significantly differed between the early and late learning periods). Together, these additional results indicate that the report trials that we measured at the very end of the fMRI experiment provide a valid proxy for the explicit learning that took place during the early learning trials.

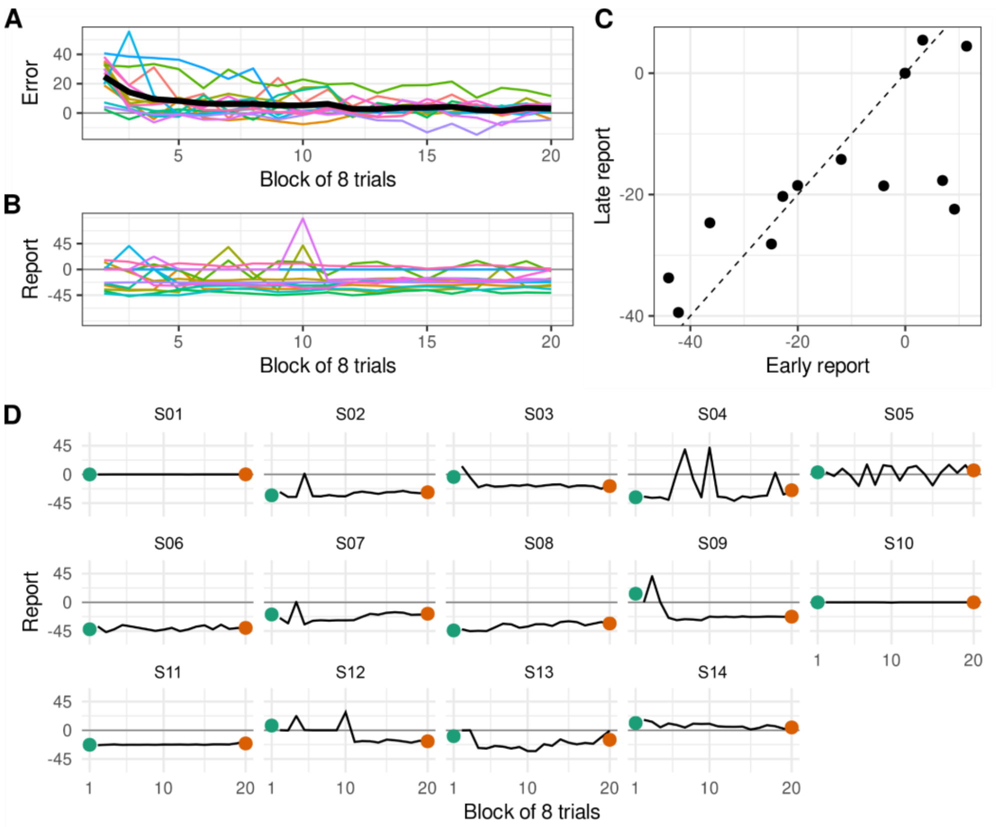

We have now included these findings as a paragraph in our Results section (see below), and as a figure in the supplement. For example, in our Results we now write the following:

"Our decision to elicit subjects' reports at the end of the learning block was based on evidence that report trials may make subjects aware of the visuomotor rotation and predispose them to use an explicit strategy during the task (de Brouwer et al., 2018; Maresch et al., 2021). Nevertheless, group-level behavioural studies suggest that subjects' explicit re-aiming strategies may evolve during learning the process (e.g., have a larger magnitude during early than late learning; Taylor et al., 2010, Taylor et al., 2014). As such, reports collected at the end of the task may not directly reflect the re-aiming strategy subjects used during the early learning process itself. To address this possible concern, we conducted a separate behavioral experiment outside of the MRI scanner (N=14), in which report trials were interspersed continuously throughout the learning block (see Supplemental Figure 1). Importantly, we found that subjects' explicit reports collected in the final trials of the learning block were highly correlated with reports collected during the early learning block ($r = 0.77$, $t_{12} = 4.22$, $p = 0.0012$). Further, we found that subjects' typically developed explicit awareness of the rotation early in the learning block, and that their subsequent explicit reports were highly stable throughout the VMR task. Taken together, these findings suggest that the late reports collected during our MRI testing session serve as a valid proxy for the explicit strategy developed during this early learning period."

DLPFC being implicated not in the explicit component but in visuomotor performance is rather unexpected. The authors propose that this makes sense given than DLPFC supports "the task-dependent updating of behaviour in response to errors" (page 10). But isn't that what the strategizing (re-aiming) of the explicit component essentially does? It is a very

delicate line whether the processes that mediate visuomotor performance (as defined here), and explicit learning are really distinct.

I think the authors addressed this nuance in the intro ("For example, explicit processes such as strategy use have been associated with the activity of prefrontal cortex [18]; however, it is unclear whether this activity reflects the use of explicit strategic processes per se or the attendant outcomes of using those processes (e.g., the resulting change in performance errors)."). My impression is that the current paper does not provide compelling evidence to conclude on this issue, and the delineation between the processes and regions underlying the performance axis and the explicit axis remain fuzzy.

Author reply:
We thank the reviewer for these comments and appreciate that we were not clearer in our delineation of the putative processes that are essential for basic task performance versus processes more directly linked to explicit learning. We have now considerably revamped our Discussion section to better describe these distinct processes and how they may map onto our neural findings. In further support of this goal, we have also included a new figure in our Discussion section (as noted in the preamble) that provides a contextualization of our findings with respect to known properties of different functional brain networks. Though we appreciate some of the reviewers' skepticism towards this alternative view, we believe it is important to also recognize that we have collected aspects of subjects' behaviour that have never been obtained before in previous neurophysiological studies (i.e., subjects' explicit and implicit components), which has allowed us to parcel out distinct patterns of neural activity associated with different facets of learning that have not been disentangled before. We believe that this novel perspective actually helps to reconcile several seemingly disparate findings across research fields (and not just those in the motor learning literature).

We have included the key aspects of this extended Discussion section (and the summary figure) below for the reviewer. We hope that these changes help to better delineate our current thinking about the present data.

"A second key feature of our results is the distinction between two separate patterns of functional connectivity during sensorimotor learning: one linked to explicit learning and the other linked to visuomotor performance. In prior work, explicit learning has been suggested to involve higher-order control regions in the prefrontal cortex, as well as processes like working memory and attention (Areshenkoff et al., 2022; McDougle et al., 2016). Yet, the specific influence of these control signals on motor system parameters has been unclear. For instance, it remains to be formalized whether these control signals dictate the selection of a new policy (i.e. a decision to aim somewhere other than directly at the target), the selection of an alternative forward model, or the weighting of specific sensory inputs based on task demands. Indeed, regions of the prefrontal cortex have been implicated in exactly these types of processes (e.g. Bédard and Sanes, 2014) and associated with performance during visuomotor adaptation (Taylor et al., 2010; Taylor and Ivry, 2012; Areshenkoff et al., 2022; McDougle et al., 2016). However, the extent to which these Control networks specifically contribute to the learning of an explicit strategy per se – versus processes which may support task performance more generally (e.g., goal-directed spatial attention), and thus lead to better performance – has not been systematically studied in previous work. Indeed, Control network areas like the DLPFC have been implicated in processes essential for basic task performance – such as the selection of spatial targets for action and with performance monitoring (Iba and Sawaguchi, 2003; Mitchell et al., 2008; Holroyd and Yeung, 2012) – in addition to any specific role in explicit learning. Our current results suggest that functional connectivity with the DLPFC (Control-A subnetwork) is not associated with explicit

learning specifically, but rather with processes that assist in task performance (e.g., spatial attention, error detection). The fact that expression of this axis was not correlated with learning rate in the separate reward-based motor task may thus reflect the fact that this axis includes (in part) patterns of connectivity associated with the unique demands of the VMR task (e.g. spatial target processes), which are not relevant to performance in the reward-based task, and/or with effects related to the monitoring of performance (e.g., detection of directional errors), rather than processes contributing to the formation of a strategy.

Although our finding that explicit learning was associated principally with regions of the default mode network (DMN) may be unexpected in the context of much of the sensorimotor learning literature, there are numerous findings across diverse research fields hinting at just such a role. For instance, recent theories of DMN functioning (e.g. Smallwood et al., 2021) have implicated the DMN in states such as self-referential processing, theory of mind, and spontaneous states such as mind wandering – all of which depend on explicit conscious attention (Mesulam, 1998; Spreng et al., 2009; Christoff et al., 2009; Andrews-Hanna et al., 2010, 2014; Smallwood et al., 2021). In addition, there is emerging neurophysiological evidence that DMN areas are uniquely engaged in conditions when performance is initially poor and when executive control is advantageous (Paulus et al., 2001; Pearson et al., 2011a). For example, work in nonhuman primates has shown that activity in DMN areas is linked to the formation of strategies during periods of learning when errors are high (Pearson et al., 2011b). Further, early neuroimaging work in explicit motor sequence learning has implicated regions of the DMN in task performance, though notably much of this work had preceded the characterization of the DMN as a distributed functional network (e.g. Willingham et al., 2002).

In this way, our results suggest that – like in multiple other domains –the DMN may play a role in motor control that is linked to the capacity of humans to explicitly attend to their own thoughts and actions. This skill is particularly important when behaviour must be guided by internally generated strategies and rule-sets, and by information accrued over longer time horizons than by in-the-moment sensory inputs (Smallwood et al., 2021). This contribution of the DMN to behavior is hypothesized to be enabled through it’s unique topographic positioning on cortex (Margulies et al., 2016), with each core node of the DMN being located maximally distant from sensory and motor systems. This topography is thought to free DMN areas from the constraints of extrinsically driven neural activity, thus allowing these regions to play a role in the orchestration of information processing across distributed functional systems (Buckner and Krienen, 2013; Smallwood et al., 2021).

Indeed, as noted in our results, we found that our three neural axes are situated at increasing positions along the cortical processing hierarchy that spans from unimodal to transmodal cortex (see Figure 7). The implicit learning axis involves regions directly adjacent to primary sensory and motor regions, consistent with the need for near instantaneous access to information encoded within sensorimotor regions, and the ability of implicit adaptation to occur automatically and outside conscious awareness (see Figure 7). By contrast, the explicit learning axis involves regions positioned at the very apex of the hierarchy, in the DMN. This is consistent with explicit learning requiring a high-level understanding of the task structure (i.e., that the cursor has in fact been rotated rather than left/right displaced), which itself requires the integration of information over the timescale of multiple trials (see Figure 7). This hierarchical view accords with functional differences in the time horizons over which sensorimotor versus transmodal areas process information (Huntenburg et al., 2018). Previous research demonstrates that temporal windows of information processing change hierarchically across the cortex (Hasson et al., 2008, 2015; Baldassano et al., 2017), with sensorimotor areas integrating information over milliseconds to seconds, and transmodal areas over seconds to minutes or longer. These longer "temporal

receptive windows" in transmodal cortex (Hasson et al., 2008, 2015) are thought to enable the encoding of more slowly changing states and the incorporation of acquired knowledge into complex scenarios, both of which are crucial for developing cognitive strategies during learning.”

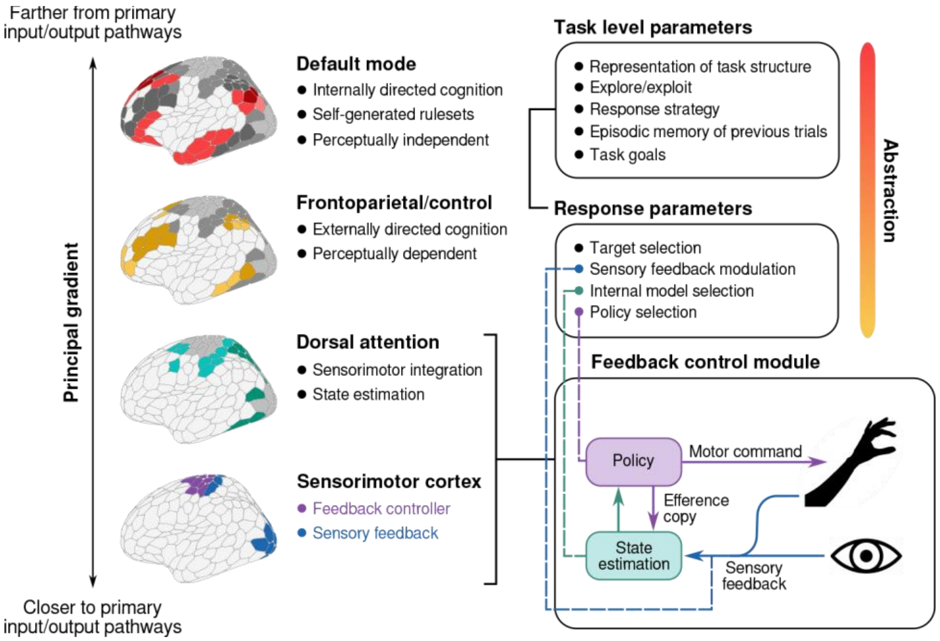

We hope that with this new and improved Discussion section that we have done a better job of conveying our interpretation of the present results.

The figure panels are not lettered, and the way the authors refer to them in the text and captions is not precise, leaving the reader with having to do the mental gymnastics to understand what is being referred to. I think this can be ameliorated. Same goes for many key elements that could be clarified (is acronym RL defined at first instance? Y-axis in figure 1 histograms is not defined. Reader has to search to find that learning rate is k in figure 6).

Author reply:
Sorry about the headache, and we appreciate these suggestions to improve the paper. We have now made all these requested changes to help improve readability and help decrease cognitive load. Thanks.

I like the negative controls used in the RL task (tongue, ipsilateral M1). I feel the authors should also perform these negative controls in the VMR task. This would address my main concern which relates to a quest for ground truth or additional validation that what comes out of these complex analyses is robust

Author reply:
We appreciate the opportunity to clarify our analyses and believe there may be some misunderstanding regarding the analyses performed for our VMR study. Our analysis employed a group-penalized ridge regression model to predict subjects’ explicit and implicit learning, as well as their performance errors. The coefficients from these separate models were then used to

create three distinct neural axes corresponding to Explicit Learning, Implicit Learning, and Performance.

It is important to note that with this regression approach, we cannot perform negative controls in the same manner as we do for the across-task prediction in the reward-based task. For instance, while it is possible to derive an 'implicit axis' using connectivity with the ipsilateral tongue area, we would of course still expect this axis to correlate well with implicit learning since the VMR task serves as the training set. This would occur despite the presumed irrelevance of the tongue area connectivity to the task. Rather, the true tests of the robustness of our derived neural axes are twofold:

1) In the case of the implicit axis, where we do not expect cross-task predictive ability, the neural substrates of implicit adaptation have already been well characterized (Hardwick et al., 2013 meta-analysis). In particular, accounts of implicit adaptation by the updating of an internal model by sensory prediction errors have already mapped out the premotor and parietal circuitry which supports such adaptation, which comprises large parts of the dorsal attention network that we define in our study. In this way, we already have a ground truth account of implicit adaptation, and it completely agrees with our derived implicit axis.
2) In the case of explicit learning, whose neural substrates have not been well characterized, we have successful across-task predictions in a separate task which is known to rely primarily on explicit processes. Further, this predictive ability is abolished when we consider connectivity with parts of the motor cortex *other* than the contralateral hand area. It is also abolished when we tested for across-task prediction (i) in the Implicit or Performance neural axes, (ii) during the pre-learning and post-learning periods of the reward-based task (i.e., our effects were selective to the learning period) and (iii) when we constructed our models using the late learning period of the VMR task.

The selectivity of these results, and the ability to generate successful predictions in a novel task, attest to the validity of our derived explicit axis. We hope the reviewer appreciates that this type of across-task prediction is quite unique in the neuroimaging literature, especially in the area of motor learning.

What is the use of defining a late learning period in the VMA task. I am missing a result pertaining to that period?

Author reply:
We apologize for this oversight on our part. We had meant to include those findings as a control analysis in our original paper (those results were accidentally deleted in the submitted manuscript file). However, we have now included these results in our revised manuscript (see our Results text below), so this hopefully reconciles this issue.

In our Results, we now write the following:

"Another alternative explanation of our main results is that Explicit neural axis, instead of reflecting processes related to the learning of an explicit strategy, relates purely to the implementation of the learned strategy (e.g., mental rotation of the response). If this were true, then we would expect to also observe the same pattern of across-task prediction effects when deriving an Explicit neural axis using instead the late learning epoch of the VMR task (the last four blocks of 8 trials). Indeed, during this late learning epoch subjects' performance has plateaued and their RTs are decreased (Fig. 1D), consistent with an increased automaticity in the motor responses. To test this idea, we again repeated the construction of the three neural axes using the patterns of functional

connectivity during the late learning epoch of the VMR task, and again found no correlation between any of these axes' expression and performance in the reward-based learning task (supplemental Figure 5A)."

The definition of the 17 networks is poorly described, especially given that results are sometimes reported at a coarser level. For instance it is not obvious what differentiates Control A, B and C. As another example, this part of caption 2 is confusing: "Note that, while we use the 17 network solution for the majority of our analyses, some of our results are reported at a coarser level (e.g. "Default mode" for Default A, B, and C). These coarse labels are, in general, nonequivalent to the 7 network solution."

Author reply:
Apologies for not providing some further context here and we appreciate why this might be confusing. The Yeo et al. 7-network and 17-network assignments of brain regions are the standards in the fMRI field, and researchers in cognitive neuroscience often report their findings with direct reference to these different brain parcellations (and/or directly parcellate the brain according to these networks, as done in our study). The 7-network and 17-network parcellations are very similar to one another, but not exactly equivalent. The division of the classic 7 network scheme (i.e., Visual, Somoatomotor, Ventral Attention, Dorsal Attention, Limbic, Control and Default mode network) into smaller subcomponents (e.g., Control A, B, C) is purely based on cluster optimizations of resting-state functional connectivity data, but there is also some evidence in the literature that the different subnetworks may subserve distinct brain functions (e.g., Control C has been particularly implicated in adaptive control functions). All of these points are well beyond the scope of the current paper — and if anything, speak to the idiosyncrasies of the neuroimaging field — but we nevertheless appreciate that some of our terminology was confusing. Rather than add more unnecessary and confusing text to our paper, we have instead opted to remove all this confusing terminology, as well as any reference to the 7-network assignment.

In Figure 5 bottom right, what does the size of dots mean?

Author reply:
Thanks for catching this — the size of the dots indicated the strength of each region's loading onto each axis. However, in the new version of the paper we have reformatted this figure in light of some of the reviewers/ comments (see the new Figure 4 below), where this is no longer an issue (i.e., the strength of loadings can now be read directly from the y-axis of the plot).

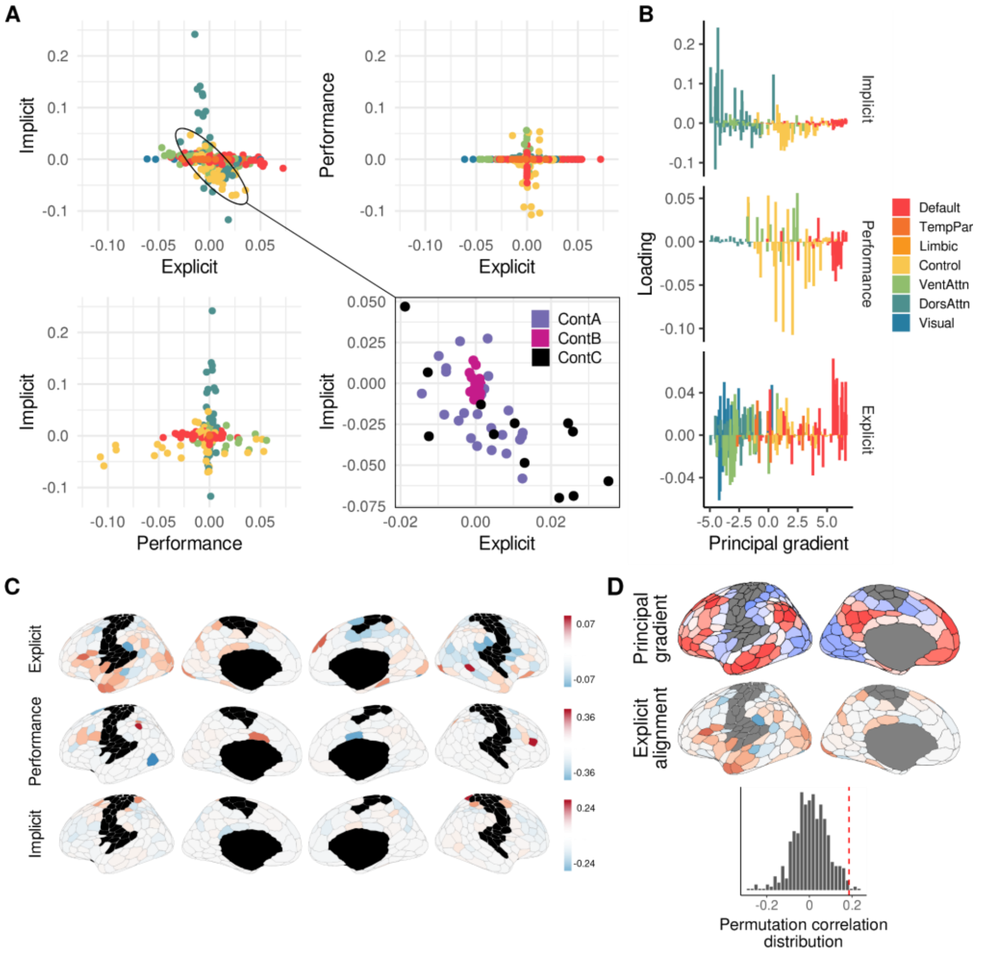

What is the exact p-value here? (page 7: When we tested for statistical significance (with Pearson's r) using a spatial autocorrelation-preserving null model ["spin test"; 34], we found that our explicit alignment neural axis, derived from our motor learning study, was significantly positively correlated (p-spin < 0.05) with this principal functional cortical gradient, derived from spontaneous intrinsic activity.). I'm asking because it seems to sit right on the .025 and .975 quantiles in Figure 5.

Author reply:
Apologies for omitting this important information. We appreciate that in the previous figure it was difficult to see the statistical significance via the bar and whiskers plot. In the new version of the figure (see panel D above), we instead indicate the statistical significance as a red vertical line amidst a null distribution. We hope this makes more sense now. In addition, we also report the p-spin results in our Results section with the following text:

"Notably, we found this explicit alignment axis was significantly correlated with the aforementioned principal gradient of functional brain organization ($p = 0.028$; spin permutation test: Markello and Misic, 2021); Figure 4 D). This suggests that explicit learning is supported by brain areas (i.e., transmodal regions) positioned at the very apex of the cortical processing hierarchy whereas implicit adaptation is supported by brain areas (superior parietal and premotor regions) located directly adjacent to sensorimotor cortex."

I'm confident that the fMRI methods are state of the art but they are written in quite a jargony manner. They are difficult to approach for non-specialists.

Author reply:
We appreciate the vote of confidence — we (obviously) agree that the methods are state of the art ;). However, with all joking aside, we also appreciate that the original version of the manuscript was a little jargony. This is important feedback for us, as we believe a great deal of the significance of our findings lies with researchers in the motor learning area, many of whom may not commonly use fMRI as part of their standard toolkit.

We have done our best to go through the paper and remove as much of this jargon as possible. We also hope that with the significant revamping of our Discussion section, and the inclusion of the new summary figure, this helps make our findings more approachable for the non-specialist. We do think that this study offers a truly novel perspective on the role of different brain networks in motor learning, and how these roles may stem directly from neuroanatomical features of their organization in cortex.

Page 4 line 1: as well as
Page 5 line 2: as an epoch
Page 5, 3 lines from bottom: it is noteworthy
Page 10 line 10: related to subjects' performance
Page 10 line 25: on our measures of subjects'
Page 11 line 9: guided by internally
Page 11 line 11: its unique
Page 19 line 26: reward-based and VMR motor tasks
Page 20 line 29: about the nature
Page 21 5 lines from bottom: As the RL task
Page 24: 4 paragraph from bottom: what about the "…"?
Page 24: 3 paragraph from bottom: a publicly available

Author reply:
Thanks. We have made these corrections.

Thanks again for your comments and feedback to help improve the manuscript.

References

Bhanpuri, Nasir H., Allison M. Okamura, and Amy J. Bastian. 2013. "Predictive Modeling by the Cerebellum Improves Proprioception." The Journal of Neuroscience: The Official Journal of the Society for Neuroscience 33 (36): 14301–6.

Chancel, Marie, Heather Iriye, and H. Henrik Ehrsson. 2022. "Causal Inference of Body Ownership in the Posterior Parietal Cortex." The Journal of Neuroscience: The Official Journal of the Society for Neuroscience 42 (37): 7131–43.

Fang, Wen, Junru Li, Guangyao Qi, Shenghao Li, Mariano Sigman, and Liping Wang. 2019. "Statistical Inference of Body Representation in the Macaque Brain." Proceedings of the National Academy of Sciences of the United States of America 116 (40): 20151–57.

Fernandez-Ruiz, Juan, William Wong, Irene T. Armstrong, and J. Randall Flanagan. 2011. "Relation between Reaction Time and Reach Errors during Visuomotor Adaptation." Behavioural Brain Research 219 (1): 8–14.

Taylor, Jordan A., John W. Krakauer, and Richard B. Ivry. 2014. "Explicit and Implicit Contributions to Learning in a Sensorimotor Adaptation Task." The Journal of Neuroscience 34 (8): 3023–32.

Tsay, Jonathan S., Hyosub E. Kim, Samuel D. McDougle, Jordan Taylor, Adrian Haith, Guy Avraham, John W. Krakauer, Anne Collins, and Richard Ivry. 2023. "Strategic Processes in Sensorimotor Learning: Reasoning, Refinement, and Retrieval." psyarxiv.com/x4652.

Tsay, Jonathan S., Hyosub Kim, Adrian M. Haith, and Richard B. Ivry. 2022. "Understanding Implicit Sensorimotor Adaptation as a Process of Proprioceptive Re-Alignment." ELife 11 (August). https://doi.org/10.7554/eLife.76639.

Zhang, Zhaoran, Huijun Wang, Tianyang Zhang, Zixuan Nie, and Kunlin Wei. 2024. "Perceptual Error Based on Bayesian Cue Combination Drives Implicit Motor Adaptation." ELife. https://doi.org/10.7554/elife.94608.1.

Reviewer #3: In this manuscript Areshenkoff and colleagues aimed to map patterns of neural connectivity to implicit and explicit motor learning processes. The question is very interesting, and the results tell a nice story, but there are multiple gaps in the methods which raises concerns regarding the reliability of the results.

1. A significant body of research over the past decade (which the authors significantly contributed to) looked into implicit vs explicit learning, suggesting various methods to measure their contribution during and after learning (reviewed by Maresch et al 2021) and methods to induce one and suppress the other. For example, delayed feedback was demonstrated to suppress implicit learning and using task-irrelevant clamped visual feedback can induce only implicit learning (Morehead et al 2017). Using such an approach and having two separate implicit and explicit tasks would have been the obvious approach to use here since while both processes are taking place there is a trade-off between them that is changing over learning and specifically during the window of early learning (first 34 trials) their contribution is changing significantly. Moreover, while learning with both mechanisms the way we measure their relative contribution is affecting learning. The reported aim is known to make participants more aware of the need to aim and increase their explicit learning.

More importantly, the biggest challenge here is that the authors didn't measure the change in implicit vs explicit, moreover, they didn't measure it at all during early learning. Their measure of explicit learning is taking place over in late learning, where typically the implicit learning component increases and the explicit decreases relative to early learning, while they are analyzing the neural connectivity during early learning (first 34 trials).

The authors note that they "focused analysis on this early learning period as this is when the contributions of explicit processes to learning are thought to be most prominent", yet their reported aim trials to measure explicit learning were over 100 trials later after learning plateau and after the implicit-explicit ratio has changed.

The author gives example participants where the more rapid learner expressed more explicit knowledge (fig1) - was it a pattern or a single example? We can assume that the more rapid learner expressed more explicit knowledge during early learning, but was it true during the reported trials after learning plateau?

I think that with the current data, since the implicit vs explicit contribution is only measured after the learning plateau, for meaningful and reliable results the window for neural connectivity must also be after the learning plateau (the last 34 trials of the learning period).

Author reply:
We appreciate this thoughtful critique from the reviewer, and we agree with much of what they have written here. We can tell that, on the one hand, the reviewer appreciates our decision to not have reporting trials occur during early learning, given the obvious concern that such reporting during early learning would bias subjects towards using an explicit strategy (a potential major issue for an individual differences fMRI study). Yet, we, like the reviewer, also recognize the shortcoming of this approach, as it does not allow us to capture the time evolution of the explicit and implicit components throughout learning. This would indeed be concerning if the explicit and implicit learning components were relatively dynamic during learning, and thus the state of the explicit component being measured at the end of learning (as we do in the current study) was not a valid proxy for the explicit component being used during early learning.

As we noted in our preamble (at the beginning of this reply document), we believe we have now addressed this potential concern by performing a separate behavioral study (N=14) in which we directly investigate the relationship between subjects' magnitude of explicit re-aiming during the early versus late learning time windows. As noted, in this study we observed a near perfect correspondence between subjects' explicit reports during these two time periods (see figure below). In addition, as shown in the single subject data, of the subjects who reported using an explicit re-aiming strategy, this strategy (1) was formed quite early during the learning period (i.e., during the early learning window) and (2) was quite stable across the full learning period (i.e., we did not find evidence that subjects' explicit aiming significantly differed between the early and late learning periods). We believe this is a crucial behavioral observation that supports our overall fMRI approach — specifically that the report trials that we measured at the very end of the fMRI experiment provide a valid proxy for the explicit learning that took place during the early learning trials.

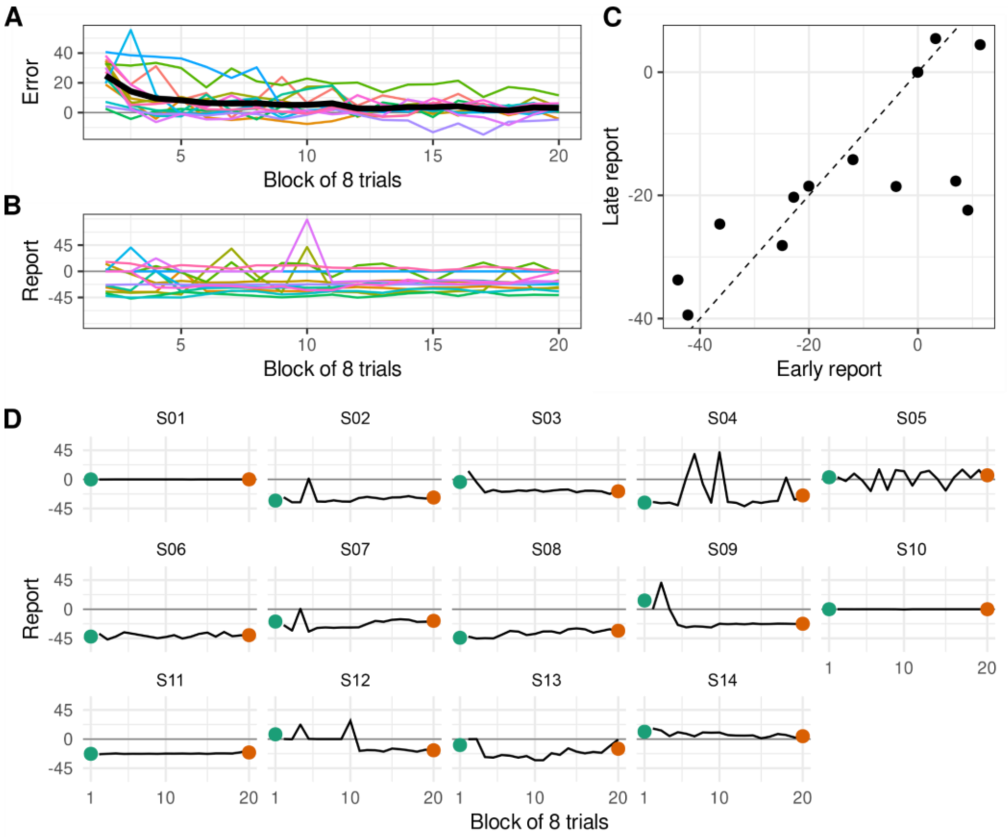

We have now included these findings as a paragraph in our Results section (see below), and as a figure in the supplement. For example, in our Results we now write the following:

"Our decision to elicit subjects' reports at the end of the learning block was based on evidence that report trials may make subjects aware of the visuomotor rotation and predispose them to use an explicit strategy during the task (de Brouwer et al., 2018; Maresch et al., 2021). Nevertheless, group-level behavioural studies suggest that subjects' explicit re-aiming strategies may evolve during learning the process (e.g., have a larger magnitude during early than late learning; Taylor et al., 2010, Taylor et al., 2014). As such, reports collected at the end of the task may not directly reflect the re-aiming strategy subjects used during the early learning process itself. To address

this possible concern, we conducted a separate behavioral experiment outside of the MRI scanner (N=14), in which report trials were interspersed continuously throughout the learning block (see Supplemental Figure 1). Importantly, we found that subjects' explicit reports collected in the final trials of the learning block were highly correlated with reports collected during the early learning block ($r = 0.77$, $t12 = 4.22$, $p = 0.0012$). Further, we found that subjects' typically developed explicit awareness of the rotation early in the learning block, and that their subsequent explicit reports were highly stable throughout the VMR task. Taken together, these findings suggest that the late reports collected during our MRI testing session serve as a valid proxy for the explicit strategy developed during this early learning period."

Next, with respect to the reviewers' second comment about using brain connectivity during the late learning window, we had actually meant to include those findings as a control analysis in our original paper (those results were accidentally deleted in the submitted manuscript file). Indeed, reviewer #2 above had caught this discrepancy in our paper.

Our original intention for including these as control results is that it would allow us to disentangle whether that the results during early learning more directly reflect the *formation* of the explicit learning strategy (which occurs during early learning period in subjects who adopt such a strategy) versus the *implementation* of that strategy (which occurs during late learning). We have now included these control results in our paper (i.e., developing an explicit neural axis based on the late learning epoch), which shows that we do not obtain any across-task prediction when using the late learning epoch. This is an interesting result, as it suggests that the explicit axis during early learning mainly reflects explicit *learning* per se (i.e., formation of the strategy), rather than simply using the already formed strategy.

In our Results, we now highlight this finding with the following text:

"Another alternative explanation of our main results is that Explicit neural axis, instead of reflecting processes related to the learning of an explicit strategy, relates purely to the implementation of the learned strategy (e.g., mental rotation of the response). If this were true, then we would expect to also observe the same pattern of across-task prediction effects when deriving an Explicit neural axis using instead the late learning epoch of the VMR task (the last four blocks of 8 trials). Indeed, during this late learning epoch subjects' performance has plateaued and their RTs are decreased (Fig. 1D), consistent with an increased automaticity in the motor responses. To test this idea, we again repeated the construction of the three neural axes using the patterns of functional connectivity during the late learning epoch of the VMR task, and again found no correlation between any of these axes' expression and performance in the reward-based learning task (supplemental Figure 5A)."

Finally, the reviewer asked about the single subject data presented in Figure. 1. These were just example participants. In the new version of Fig. 1, we have instead opted to show the distributions of learning parameters across all participants (see below), so that the reader is not incentivized to extrapolate from single subject patterns to all other subjects. In any case, it is indeed true that subjects who exhibit a larger explicit component also tend to exhibit lower error during late learning (Fig. 1D), as well as higher RTs (consistent with the time required to implement the re-aiming strategy; Fig. 1D, two right panels).

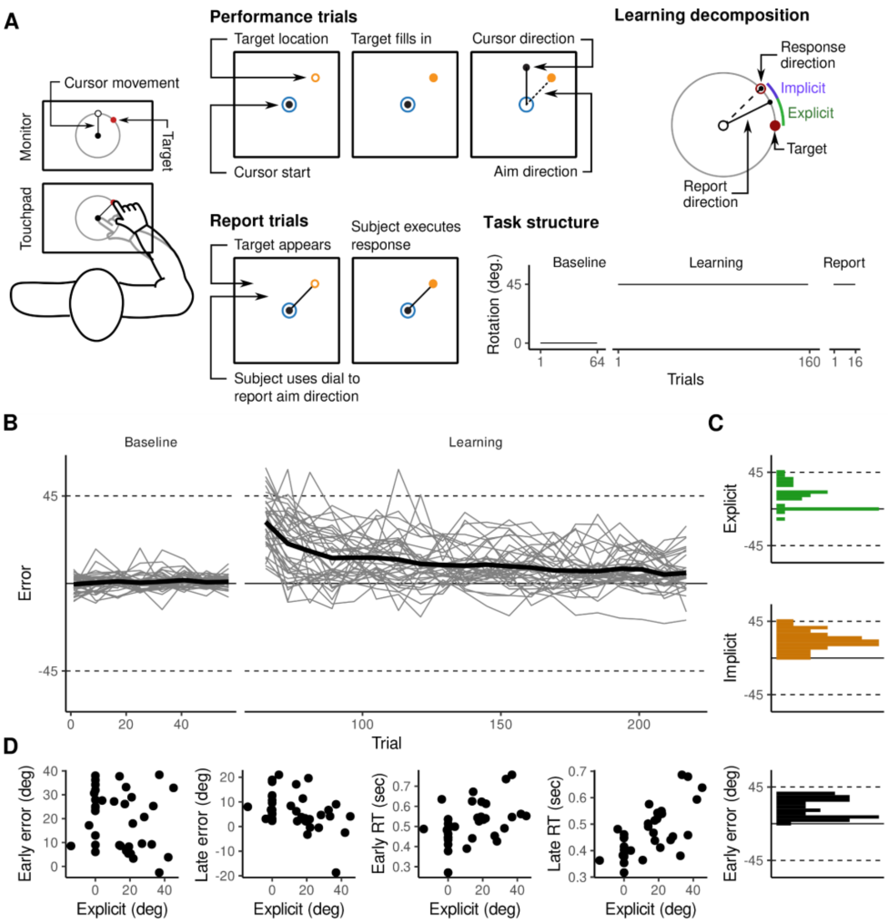

We hope that with these new behavioral and neural additions to the paper that the reviewer is better convinced that the Explicit neural axis defined from the early learning window is indeed related to subjects' explicit learning.

2. In their second task the authors seem to mix reward learning and explicit learning. Reward-based learning can be implicit. Actually, the paper the authors cite as a reference for reward-based learning being explicit is titled "Implicit reward-based motor learning" and demonstrates that even while learning from binary reward there is an implicit component , let alone learning with scalar reward feedback as was done here. The "abrupt periods of rapid learning" or "Aha! moments" are indeed explicit, but this is also common in VMR (and even more so in VMR with aim reporting) so it's not clear why the authors took this approach around the "Aha! moment" here and not in the VMR.

Interestingly, the gradual improvement (which the author used to define a learning rate) suggests that this wasn't an abrupt improvement, but a gradual one as seen in the example participants in Figure 6 and most participants in Figure 7.

Author reply:
We appreciate the opportunity to clarify these points. We agree that reward-based motor learning can be implicit — however, our general point about this was that such learning is predominantly explicit. Indeed, we cited the paper "Implicit reward-based motor learning" because it makes the very point that the implicit component to reward-based motor learning is really quite small, and

that the majority of reward-based motor learning is explicit. There are several other studies, which we also cite, that also support this general contention (Holland et al., 2018; Codol et al., 2018, De Brouwer et al., 2022; and the study of Dam et al., 2013, which helped provide motivation for the current study).

As for the second point, we completely agree that the abrupt periods of rapid learning are indeed explicit learning moments during the reward-based task, and that these can also occur during VMR. However, given the nature of the instantaneous visuomotor rotation and the resulting large, immediate errors experienced by participants, the 'Aha moments' during the VMR task overwhelmingly take place during the early learning window, and are thus already captured by our analyses. [Note that it would also be potentially challenging to look for these 'Aha moments' beyond this early learning window, as in principle they would be superimposed atop a growing implicit learning component, which is prominent during VMR tasks]. By contrast, in the reward-based task, the 'Aha moments' are quite variable — and often readily observable — across subjects, hence why we resorted to our sigmoid function fitting procedure to identify these time windows in a purely data-driven fashion.

Finally, re: the reviewer's comment about the gradualness of these learning periods, it is true that these are not 'single-trial' learning periods, and thus our usage of the term Aha moments might be confusing or misinterpreted by readers. We have thus removed all reference to 'Aha moments' throughout the paper, and have instead rephrased our description of these moments as "clearly defined learning periods". Please see the main text for these changes.

Minors

1. In Figure 1 the explicit and implicit distribution don't seem to complete each other — a cleaner visualization of explicit implicit and overall learning across the group would be helpful.

Author reply:
We have now included a figure panel showing the full distribution of implicit and explicit scores for all subjects. Note that, as subjects do not necessarily fully adapt by the end of the learning block (that is, they do not in general achieve exactly zero error), the implicit and explicit scores do not necessarily sum to 45 deg. (to full adaptation). In particular, there is considerable variability in the degree of adaptation, and in how much of this adaptation is accounted for by explicit/implicit learning. We hope that this is clearer.

2. The lack of numbers on the axes makes it difficult to understand and interpret the results. E.g. in Figure 4, in the 3D projection and the top 2D projection it seems like the control areas are spreading along multiple axes while from the following 2D panels it seems to be mostly along the performance axis. Is the scaling on the three 2D plots identical? The 3D plot is with different scales over the axes also making it difficult to interpret.

Author reply:
We appreciate the reviewer pointing this out to us. The reviewer is correct that the scaling of the three 2D plots was not identical in the previous version of the manuscript, and we have now rectified that, as well as changed the figure layout, in the newest version. We have included our newest figure below. We hope that this new figure makes it clearer that the Control subnetworks — specifically the A and C networks — are exhibiting differential loading along the implicit and explicit neural axes.

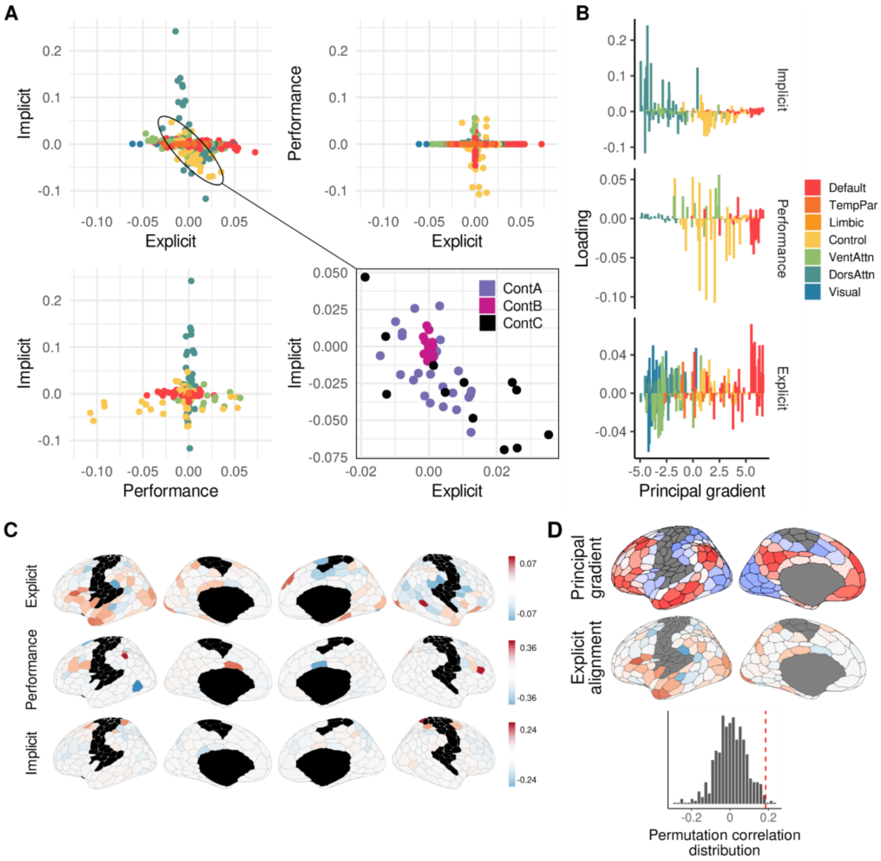

3. Please add numbers/letters to the figure panels. It's difficult to match the reference in the text to the right panel.

Author reply:
Reviewer #2 noted this same concern with the figures. We have now updated the figures to include letter subpanels, reducing the mental gymnastics required to interpret what is being shown. Thanks!

Maresch, J., Mudrik, L., & Donchin, O. (2021). Measures of explicit and implicit in motor learning: what we know and what we don't. Neuroscience & Biobehavioral Reviews, 128, 558-568.

Morehead, J. R., Taylor, J. A., Parvin, D. E., & Ivry, R. B. (2017). Characteristics of implicit sensorimotor adaptation revealed by task-irrelevant clamped feedback. Journal of cognitive neuroscience, 29(6), 1061-1074.

Thanks again for your comments and feedback to help improve the manuscript.
