# Supplementary material for: Distinct patterns of connectivity with the motor cortex reflect different components of sensorimotor learning
Source: PLoS Biol. 2024 Dec 3;22(12):e3002934. doi: 10.1371/journal.pbio.3002934 (PMC11644839; doi:10.1371/journal.pbio.3002934)
Supplement: Attachment — Submitted filename: Plos Biology reply2reviewers 2.pdf [file pbio.3002934.s009.pdf]

We thank the reviewers for their time in reviewing our manuscript and for their helpful comments and suggestions. We were pleased to hear that reviewers 1 and 2 were completely satisfied with our previous revisions. Below, we now constructively address the remaining comments from reviewer 3.

We hope that you now find the manuscript suitable for publication.

Throughout our response, we will use the following format:

(1) black text in boldface is used when we directly quote the reviewers;
(2) black plain text denotes excerpts from the original manuscript;
(3) blue text is our response; and
(4) red text is used when quoting from the revised manuscript.

Reviewer 1 :
Thank you for addressing my concerns. I have no additional comments.

Author reply:
We are happy to hear that we have adequately addressed your comments. Thanks!

Reviewer 2:
The authors have carefully revised the manuscript according to the reviewers comments. I think it is now suitable for publication. It is a solid paper. Good job.

Author reply:
We are happy to hear that we have adequately addressed your comments. Thanks!

Reviewer 3:
In this revised manuscript, Areshenkoff and colleagues try to address the methodological gaps pointed out in the previous review. While I still think the question is very interesting, and the results tell a nice story, I'm not convinced by some of the answers and the key gaps aren't resolved, hence the concerns regarding the reliability of the results remain.

1. I find the suggestion that explicit reports were "highly stable throughout the VMR task" highly controversial as it contradicts the entire literature on implicit and explicit learning. Looking into the "stable explicit component" in the new behavioral experiment, it seems like it's not exactly the case. Some participants (e.g. S13) show the expected explicit curve, increasing fast and decreasing slowly after. Some showed no explicit aim at all, some very noisy curves, and only a few showed this unexpected stable aim. While all together it leads to a significant correlation, one should be careful not to overinterpret it. Specifically, as we see here different subgroups of explicit learners. Previous studies have addressed such subgroups and related them to the RTs (e.g. Bromberg et al., 2019, eNeuro). This nicely links to the suggestion by Reviewer 1 to look at the increase in RT from baseline to early learning as a proxy measure of explicit strategy in the early learning phase. While I agree with the authors' concern that RTs correlate with other cognitive variables, it is probably the most direct way to address explicit strategy in this dataset. A curve of the change in RT over trials would be very informative and if it supports the authors' suggestion, that the explicit component is stable, they'll have an argument to justify their approach. However, the higher correlation between the reported aim and RTs during late learning (relative to early learning) suggests that the explicit strategy changes (as expected).

Author reply:
We disagree with the reviewer's interpretation of the results of the new behavioral experiment. Note that most subjects, after an initial period of variability, show relatively constant reported aim directions. Indeed, even in cases where these reports are variable (e.g., as in our subject S05), they are largely stationary.

Importantly, the suggestion that the RTs of explicit learners should be relatively stable over time contradicts the results of the previous literature — e.g., the Bromberg et al, 2019 eNeuro study cited by the reviewer. Indeed, in this very study, Bromberg et al. show that RTs decrease over time for *both* implicit and explicit learners. This is to be expected, since early trials involve the start of a new task block, with new feedback (and thus involve 'task-switching' effects), and because speed increases as automaticity is developed throughout learning. A more direct

indicator of explicit strategy use is given by the relationship between RT and accuracy (visuomotor errors), since explicit learners often use mental rotation strategies, and consequently, longer RTs are needed to perform the full mental rotation of the response direction (e.g., McDougle et al., 2019, *Nature Communications*).

Guided by this recent work and prompted by the reviewer's query, we have now included an additional analysis in our main manuscript examining the relationship between RT and accuracy in both explicit and implicit learners (see Fig. 1D below). Note that explicit learning shows a monotonic decrease in error with longer RTs, while the relationship is basically flat in implicit learners. However, we should note that this analysis is not entirely perfect: For one, since some explicit learners are known to use response caching strategies, which present with a different RT/error relationship, the actual relationship between RT and accuracy may vary, even among explicit learners. Examining the relationship between RT and error at the very beginning of the task block is also challenging, as there are too few trials to perform a detailed quantile analysis, and the initial handful of trials can be expected to have both extremely long RTs and large errors in all subjects. Nevertheless, our analysis clearly shows that the performance of explicit learners as a group, across the entire task block, is consistent with the use of a mental rotation strategy.

In our Results section, we now describe these RT results with the following text below and corresponding change to Figure 1 (also below):

"As an additional validation of our explicit reports, we further examined the relationship between subjects' error and their RTs. Explicit strategies in the VMR task often make use of mental rotation, and so lower errors are typically observed with longer response times (McDougle and Taylor, 2019). For each subject, we computed the mean error during the learning block in each of 5 bins, defined using the quantiles of their response time distribution (Figure 1D). Using these bins, we computed the (Spearman) correlation between error and RT for each subject, and observed a significantly lower (negative) average correlation within explicit (compared to implicit) learners (mexp = −0.44, mimp = 0.11, $t(34) = -2.38$, $p = 0.033$). That is, explicit learners were more accurate at longer response times, while the accuracy of implicit learners did not vary with response time."

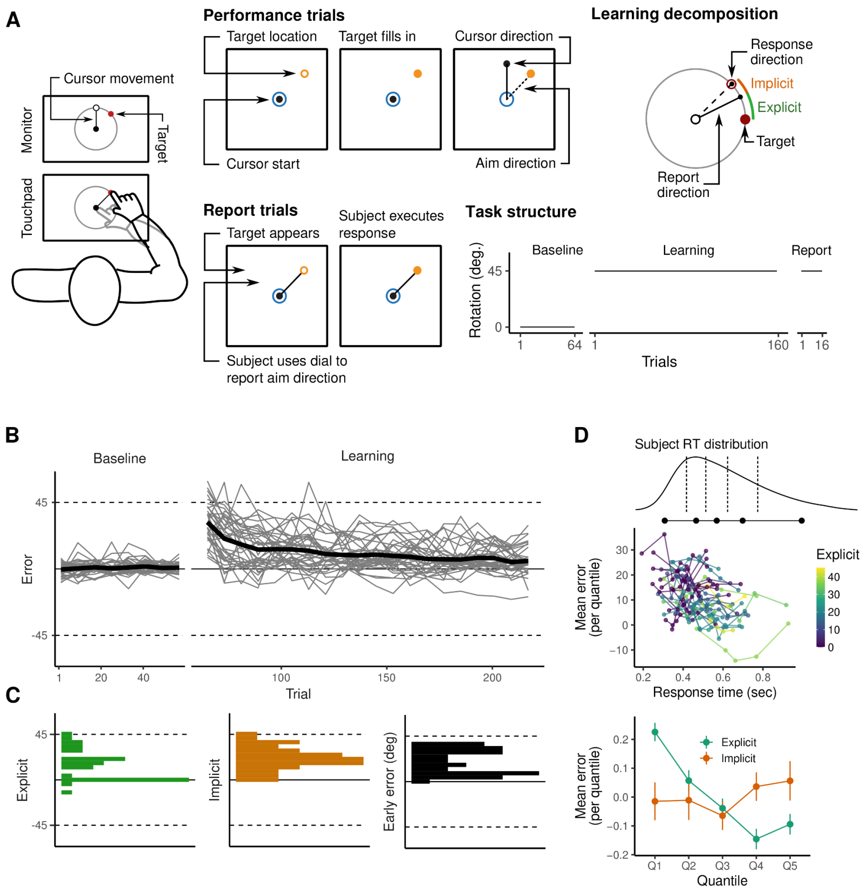

Figure 1: Visuomotor rotation (VMR) task and subject behaviour –A) Subjects were cued to move a cursor to a target appearing on a circular ring using an MRI compatible touchpad. During learning trials, the cursor moved at a 45◦ angle relative to the hand, so that subjects had to learn to adjust their movement direction in order to contact the target successfully. After learning, subjects performed a set of 'report' trials in which they used a dial to indicate their aim direction prior to executing the movement. Using these report trials, we derived estimates of subjects' total explicit and implicit learning during the task. (B) Error curves for individual subjects. Black trace in bold denotes overall mean error across subjects. (C) Distributions of subjects' estimated explicit (top) and implicit (middle) contributions to performance, as well as early error (bottom; defined as the mean error during the first 32 trials). D) For each subject, we computed the mean error during the learning block in each of five response time bins separated by the 20%, 40%, 60%, and 80% percentiles. Consistent with previous literature suggesting the use of mental rotation strategies by explicit learners (McDougle and Taylor, 2019), subjects who reported a non-zero re-aiming direction showed monotonically decreasing error with longer response times.

2. Implicit learning kicks in slowly, hence, any correction in early trials must be mostly attributed to the explicit strategy, to the extent that the performance during early learning is probably a better proxy of the explicit strategy used there than the explicit aiming reported at the end of the learning. Hence one can argue that the performance captures the early explicit strategy and the "pure" explicit after correcting for performance captures the change in explicit over learning.

Author reply:
Indeed, we agree that explicit learning is likely to be the dominant contributor to (or lack of) early learning performance. However, as noted in our paper, this presents a challenge — the neural contributions to the development of an explicit strategy are necessarily entangled with neural responses linked to error and conflict. Indeed, this was our very motivation for partialling out subjects' performance, and deriving a performance neural axis separately. We suspect (and speculate in our Discussion) that many features of the Performance neural axis — such as the involvement of the dlPFC — are likely to be essential for the implementation of a strategy as well; however, because these structures are also involved in processes purely related to performance (e.g. the selection of a spatial target for action), they are also likely to be essential even for implicit learning. For this reason, we interpret our corrected Explicit neural axis as reflecting the brain systems selectively involved in the *formation* of a re-aiming strategy, rather than the complete network supporting all facets of explicit *performance*.

3. While I agree with the authors that the network during the formation of the explicit learning strategy (during early learning) might be more interesting than that of the implementation of that strategy (during late learning), I think the latter is still interesting and I'm convinced that it will provide a far more meaningful and reliable result as it will directly relate to the measured explicit aim. In the revised manuscript the authors performed this analysis (on the last four blocks of 8 trials) but didn't share the results as they did for early learning (in figures 3 and 4) but only the across-task prediction which is difficult to appreciate without knowing if and how different the networks are.

Author reply:
We thank the reviewer for catching this, and we agree that the inclusion of the late learning neural axis would be of potential interest to readers. We have now included a detailed characterization of the Explicit axis extracted during the late learning period of the VMR task (see Supplementary Figure 6 below). Note that this axis is substantially different from the Explicit axis extracted during the early learning phase, and we have included additional commentary (in our Results) speculating as to the meaning of these differences. In particular, we note that the late Explicit axis comprises regions implicated in mental rotation, and suggest that this axis may reflect the *implementation* of a learned re-aiming strategy, rather than the formation of the strategy itself, which emerges during early learning.

In our Results section, we now write the following:

"Note also that the explicit axis derived from subjects' late learning periods was markedly different from its early learning equivalent (Supplemental figure 6B and 6C). During late learning, the explicit axis prominently featured regions of the dorsal attention and visual networks – in particular, premotor and posterior parietal regions, as well as central and peripheral visual regions. Although we do not directly assay the specific strategy used by explicit subjects, mental rotation strategies are commonly used by explicit learners in the VMR task (McDougle and Taylor, 2019), and regions of the premotor, posterior parietal, and visual cortex are frequently implicated in mental rotation (Mourao-Miranda et al., 2009). This interpretation would be consistent with the failure to observe a correlation between the expression of the late explicit axis and performance in the reward-based learning task, as mental rotation is unlikely to support learning in the reward-based task. Thus, we interpret this late explicit axis as reflecting the implementation of a strategy specific to the VMR task (e.g., mental rotation), while the early explicit axis likely involves components related to the learning of a strategy itself."

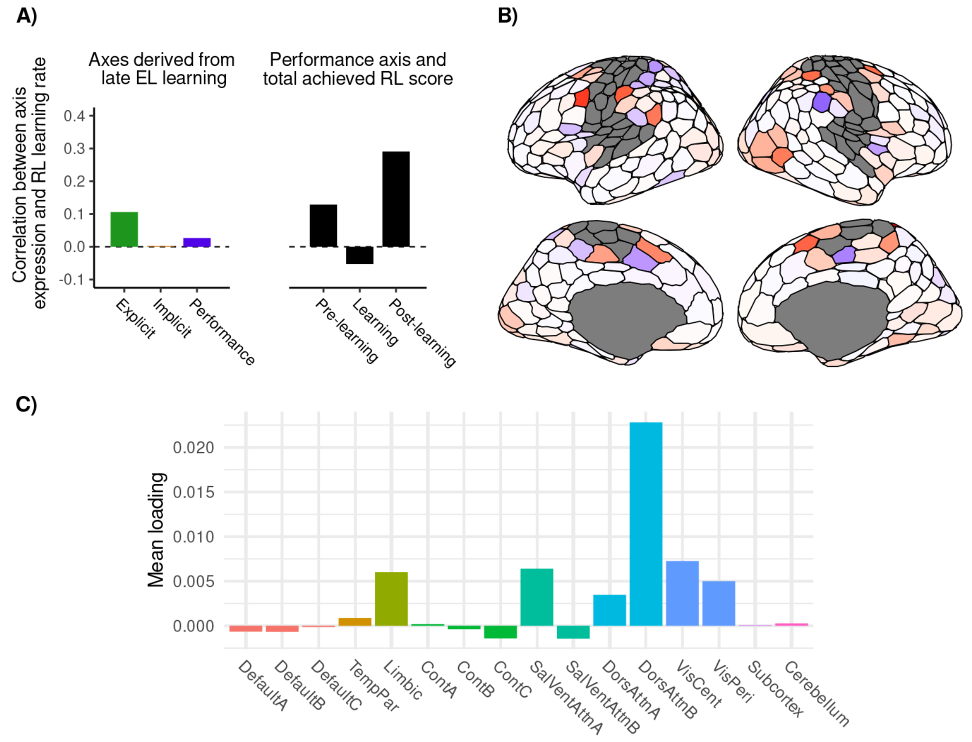

Figure 6: Explicit axis derived from late EL learning – A) We rederived each axis featured in our main analysis using the equivalent late learning period (final 100 imaging volumes) of the EL task. In no case did expression of these axes correlate with learning during the reward-learning task. We do note, however, a moderate (but non-significant) correlation between the (early learning) performance axis and the mean score achieved in the reward-based motor learning task, suggesting that the performance axis may, in part, reflect processes involved in the monitoring and evaluation of current task performance. B) Surface map of the late learning explicit axis. C) Mean loading per network for the late learning explicit axis.

4. While the authors justify their 100-volume choice based on previous work, I find the fact that this number of volumes is 34 trials slightly concerning. Having blocks of 8 trials it would make more sense to look at 32, which corresponds to 4 complete blocks (as they have done for the control analysis during late learning) or 40 trials for 5 blocks.

Author reply:
We very much appreciate the reviewer catching this discrepancy and the opportunity to clarify our methodology. Their comment has not only led us to address (and correct) this important point but also prompted us to conduct additional analyses that further bolster our findings.

First and foremost, in our original manuscript we incorrectly stated that we used 34 trials for our analysis, when in fact we used 32 trials. As the reviewer correctly noted, 32 trials correspond to 4 complete blocks, which indeed provides a more logical analytical approach and the one we used. We apologize for this oversight and have corrected this information throughout the manuscript.

Regarding the 100-volume window choice, we acknowledge the reviewer's concern and have conducted a comprehensive sensitivity analysis to assess the robustness of our effects across various window sizes. As noted, our 100-volume window size was chosen for direct

comparability with our previous work (Areshenkoff et al, 2022, Elife), but we have now replicated our main analysis using a range of window sizes, from 50 volumes to the full 100 volumes used in the primary analysis. It's worth noting that longer window sizes were not feasible due to the limited duration of several subjects' learning periods in the reward-based motor learning task. For each window size, we re-derived the three axes from the initial learning period of the VMR task and examined their correlation with learning rate during the reward-based task.

The results of this sensitivity analysis are now included in Supplementary Fig. 5 of our revised manuscript (included below). Importantly, we found that the correlation between the explicit neural axis and reward-based task performance remains consistently positive across the majority of window lengths. This analysis also demonstrates that our choice of the 100-volume window was not arbitrarily favorable to our hypothesis. Indeed, the 100-volume window we initially selected does not even represent the highest correlation value observed; specifically, the peak correlation value was actually found using a window length of 91 imaging volumes. In any case, there is strong motivation to use longer window lengths, wherever possible, given that the estimation of functional connectivity degrades in a monotonic fashion with the length of time series data used to estimate that functional connectivity (a natural consequence of correlation estimations).

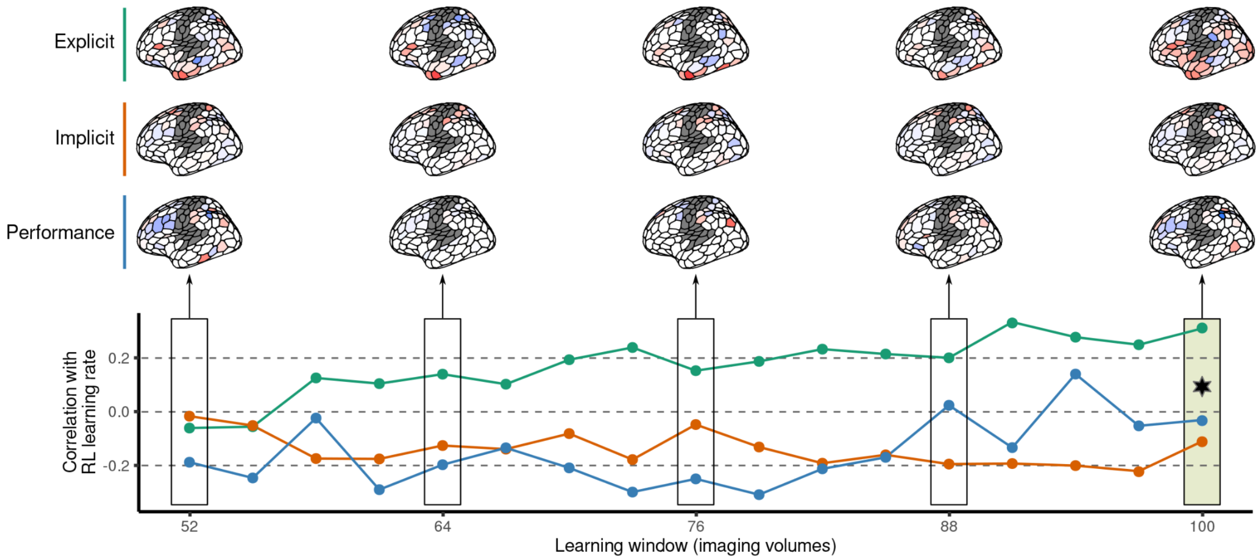

We believe this additional analysis not only addresses the reviewer's concern but also strengthens the validity and generalizability of our findings. We have updated our manuscript to include these results and their implications, providing a more thorough and nuanced presentation of our research.
